# The current regenerative medicine approaches of craniofacial diseases: A narrative review

**DOI:** 10.3389/fcell.2023.1112378

**Published:** 2023-02-28

**Authors:** Elahe Tahmasebi, Mehdi Mohammadi, Mostafa Alam, Kamyar Abbasi, Saeed Gharibian Bajestani, Rojin Khanmohammad, Mohsen Haseli, Mohsen Yazdanian, Peyman Esmaeili Fard Barzegar, Hamid Tebyaniyan

**Affiliations:** ^1^ Research Center for Prevention of Oral and Dental Diseases, Baqiyatallah University of Medical Sciences, Tehran, Iran; ^2^ School of Dentistry, Tehran Branch, Islamic Azad University, Tehran, Iran; ^3^ Department of Oral and Maxillofacial Surgery, School of Dentistry, Shahid Beheshti University of Medical Sciences, Tehran, Iran; ^4^ Department of Prosthodontics, School of Dentistry, Shahid Beheshti University of Medical Sciences, Tehran, Iran; ^5^ Student Research Committee, Dentistry Research Center, Research Institute of Dental Sciences, Dental School, Shahid Behesti University of Medical Sciences, Tehran, Iran; ^6^ Student Research Committee, Qazvin University of Medical Sciences, Qazvin, Iran; ^7^ Department of Clinical Sciences, Faculty of Veterinary Medicine, Lorestan University, Khorramabad, Iran; ^8^ Department of Science and Research, Islimic Azade University, Tehran, Iran

**Keywords:** regenerative medicine, mandible, maxilla, disease, craniofacial

## Abstract

Craniofacial deformities (CFDs) develop following oncological resection, trauma, or congenital disorders. Trauma is one of the top five causes of death globally, with rates varying from country to country. They result in a non-healing composite tissue wound as they degenerate in soft or hard tissues. Approximately one-third of oral diseases are caused by gum disease. Due to the complexity of anatomical structures in the region and the variety of tissue-specific requirements, CFD treatments present many challenges. Many treatment methods for CFDs are available today, such as drugs, regenerative medicine (RM), surgery, and tissue engineering. Functional restoration of a tissue or an organ after trauma or other chronic diseases is the focus of this emerging field of science. The materials and methodologies used in craniofacial reconstruction have significantly improved in the last few years. A facial fracture requires bone preservation as much as possible, so tiny fragments are removed initially. It is possible to replace bone marrow stem cells with oral stem cells for CFDs due to their excellent potential for bone formation. This review article discusses regenerative approaches for different types of craniofacial diseases.

## 1 Introduction

Traumatic or oncological resection or congenital abnormalities can cause craniofacial defects (CFDs). A non-healing composite tissue wound is caused by soft tissue deficits, bone loss, or both. Treatment of craniofacial defects is difficult because there are many different types of tissues and structures (Nyberg et al., 2017). Maxillofacial (MF) fractures result from many factors, such as crashes in motor fights, firearms, vehicles, wars, and sports (Herford, 2017; Castro-Espicalsky et al., 2020). Those with musculoskeletal dysfunctions usually suffer significant negative effects from CFDs (Martín-Del-Campo et al., 2019). Iran had to provide prolonged medical care to over 400 thousand patients due to the Iraq–Iran war. Official organizations authorized to assist war victims invited international reconstruction surgeons to Iran. The leader of this team was Paul Tessier, the founder of craniofacial surgery. During this project, patients with severe trauma injuries in the lower part of the face were provided with MF and oral rehabilitation using the most current techniques (Simon et al., 2015). There are differences in the incidence rates of trauma according to countries worldwide. Trauma is one of the top five factors contributing to death worldwide (Herford, 2017). United States children suffer from it more often than any other disease. Based on the National Trauma Databank collected, pediatric trauma cases most commonly involve the head, with the face being the fourth most common body part to be injured. Case fatality rates of 3.74% and 3.07% were reported for pediatric head and face injuries in 2016 (Braun et al., 2017). The burden of oral diseases is heavily influenced by periodontal disease (Latimer et al., 2021). According to the World Health Organization (WHO), the number of people affected by this high-impact disease grew by 6% from 2017 to 2018 (Global and regional, 2018). A child is likely to die from pediatric trauma and present to an ED every year; fractures occur in 8%–15% of cases of pediatric facial trauma after sustained force; over 11,000 children die each year from pediatric trauma, and over eight million children visit the ED due to pediatric trauma (Andrew et al., 2019; Rogan and Fang, 2021). There were 1,146 patients with facial fractures treated at the SDM craniofacial unit for 10 years, from January 2003 to December 2013; among them, 68 were young children (Ghosh et al., 2018). A total of 599 men and 735 women were involved in one study; 66% of patients had more than one comorbidity, and the mean age was 79.3 years. Most injuries were caused by falls, and the most commonly observed injuries were zygomatic fractures. The number of patients with concurrent injuries was 27.3% (Brucoli et al., 2020). There were 4,783 craniomaxillofacial (CMF) injuries among the 2,014 injured service members of the United States service members injured in conflicts with Iraq and Afghanistan, of which 26% had injuries to CMF regions (Lew et al., 2010). Between 2003 and 2011, all patients with facial defects and problems referred to a United States- or United Kingdom-based MF trauma that were alive were examined in the Joint Theatre Trauma Registries. In total, 16,944 casualties suffered facial injuries, and the most common problem was bone fractures and muscle/skin injuries. Inner/middle ear injuries and injuries to the teeth also occurred in large numbers (Breeze et al., 2019). During World Wars I and II, tantalum and acrylic cranioplasty were developed as a result of battlefield medicine during the Crimean War and the American Civil War. With more durable protective armor, improved medical evacuation, and early “far-forward” neurosurgical treatments available in modern conflicts such as Afghanistan and Iraq, cranial injuries are more likely to be survived (Bonfield et al., 2014; Saadi et al., 2018). Because tissues are tissue-specific and anatomically complex in that region, CFDs can present many challenges during treatment. Clinicians, scientists, and engineers can create personalized CFDs using 3D printing. These technologies can be used to restore the appearance and function of patients using three main strategies: reconstruction, rehabilitation, and regeneration (Nyberg et al., 2017). Hyaluronic acid (HA) is one of the human body components. In recent decades, HA in bone regeneration has gained widespread acceptance and is an increasingly popular topic in craniofacial surgery and dentistry. The demand for regenerative bone therapy has improved considerably during the past years, from maxilla augmentation to craniofacial bone fractures (Tahmasebi et al., 2020; Yazdanian et al., 2020; Zhai et al., 2020; Yazdanian et al., 2021a; Yazdanian et al., 2021b; Hakim et al., 2021; Soudi et al., 2021; Yazdanian et al., 2022). In addition, additive manufacturing (AM) refers to the process by which layers of materials are deposited in layers according to a design generated by computers. There has been a paradigm shift in manufacturing at the individual patient level with the advent of AM, which is a concept presented as a paradigm shift in the manufacturing of previously mass-produced biomaterials for both soft and hard tissue defects caused by congenital or acquired pathologies, periodontal disease, and MF trauma (Latimer et al., 2021). Besides this, MSCs can turn into multiple types of cells. Several dental tissues contain MSCs, including the periodontal ligament, the dental follicle, the dental pulp, the apical papilla, and the deciduous teeth. The future of regenerative medicine (RM) lies in tissue regeneration or developing new structures such as those found in craniofacial structures. This will enable the treatment of diseases such as congenital malformations and traumas (Maxim et al., 2015). This review article discusses the current approaches of regenerative therapies in craniofacial diseases.

## 2 Craniofacial and dental diseases

Craniofacial dystrophy (CFD) may be one of the underlying factors causing malocclusion. Craniofacial syndromes arise from two factors: cleft palates and craniosynostoses. Surgical techniques vary according to the particular issues to be addressed. Cranial synostosis is a congenital disease that occurs when one or more cranial sutures merge prematurely (Buchanan et al., 2014; Mew, 2014). A step-by-step morphological and cellular process governs the reciprocal epithelial–mesenchymal interactions that drive tooth growth (Balic, 2018). In addition to many other oral illnesses, pulpitis, periodontitis, dental caries, and CF trauma are the most frequent. Some people are born with CFDs or oral cancer. Dental problems are not a trivial matter, especially when there is a connection between oral and systemic illness. Patients will inevitably lose tissue due to most oral conditions, and the goal of dental therapy is to restore craniofacial function and tissue healing (Xu et al., 2021). Malocclusion and acquired craniofacial dysmorphology are caused by chronic oral dysfunction and orofacial myofunctional disorder. Getting long-term facial stability requires understanding the underlying causes of malocclusion, open bites, and hard palate collapses (D’Onofrio, 2019). The Bardet−Biedl syndrome (Visscher et al., 2016) is a congenital disease of the craniofacial area. This autosomal recessive non-motile ciliopathy is a monogenic disorder. Dental malformations are common in people with BBS because of aberrant jaw and teeth development during embryonic life. Dental care is complicated by genetically encoded intrinsic oral anatomical defects, resulting in orofacial comorbidities and diverse BBS clinical presentations. A broad range of collateral oral diseases may result from the comorbidities of the BBS phenotype, including diabetes, renal dysfunction, obesity, sleep apnea, cardiovascular dysfunction, and cognitive impairments (Panny et al., 2017).

### 2.1 Mandible and maxilla diseases

Despite its rarity, osteomyelitis of the maxilla may occur even in immunocompetent individuals due to the solid vascular supply of the maxilla. The fungus *Rhizopus*, *Rhizomucor*, and *Cunninghamella* of the Mucoraceae family are responsible for mycosis of the tongue, nose, and paranasal sinuses. In 1885, Paultauf was the first to identify mucormycosis as an illness in humans. Mucormycosis is an opportunistic fungal disease caused by saprophytic fungi (Phycomycetes) (Srivastava et al., 2019). Paresthesia of the naso-facial area, periorbital cellulitis, rhinorrhea, rhinorrhea with and without fever, nasal crusting, stuffiness and epistaxis, arthralgia, and weight loss are some of the early symptoms, followed by eschar formation and necrosis (Ferguson, 2000). Ablative surgery is necessary in most oral and paranasal sinus cancer cases, combined with adjuvant therapy. Reconstruction of significant post-ablative anomalies in the mandible and maxilla poses numerous challenges to the reconstructive surgeon. Functional and aesthetical restoration can only be achieved when doctors are intimately familiar with the underlying disease process, have a clear understanding of head and neck anatomy, and can select suitable tissues for each patient (Likhterov et al., 2019). Osteosarcomas of the jawbone are uncommon. Unlike long bone sarcomas, the patients with this condition are typically older, and metastasis rarely occurs, indicating a distinct pattern of activity compared to long bone sarcomas. According to ElKordy et al. (2018), 21 osteosarcoma cases were examined between 2006 and 2013. A total of 6 cases affected the maxilla, and 15 affected the mandible. The second instance of a periosteal response to solar radiation was observed. Four patients had improper biopsy results when the final pathology reports were reviewed for surgical resection. More commonly found among people in their late twenties, soft tissue sarcomas such as myxofibrosarcoma are called myxofibrosarcomas. It typically occurs in the subcutaneous soft tissue in the extremities, with a high likelihood of recurrence at the original site. MFS in the head and neck are rare and uncommon to have MFS of the maxilla and mandible (Li et al., 2020).

### 2.2 Cancer

In the United States, oral cancer ranks sixth in terms of incidence, and it is also the sixth most fatal. Most oral cancers are squamous cell carcinomas. An invasive biopsy, biochemical studies, and a comprehensive clinical examination can detect oral malignancies. Biomarkers, such as those found in blood, urine, and saliva, may help diagnose diseases early. Saliva offers a promising tool for early cancer diagnosis that is easy and non-invasive because it can be collected from a patient without invasive procedures. Besides proteins, peptides, electrolytes, organic and inorganic salts, and complementing contributions from gingival crevicular fluids and mucosal transudates, whole-mouth saliva contains many other biological elements (Khurshid et al., 2018). The risk of lip and oral cavity cancer has been linked to potentially malignant oral diseases (OPMDs) (Warnakulasuriya et al., 2021). Brain metastases that have spread to the breast induce receptor-mediated signaling cascades. In turn, the inward currents would be stimulated in the malignant cells. Subsequently, the tumor would grow. The brain-metastasizing cells also express neurotransmitter receptors and extend perisynaptic processes to receive neurotransmitter signals (Monje et al., 2020). Neurotransmitters and growth factors derived from peripheral nerve fibers modulate the course of gastric, pancreatic, oral, prostate, colon, and breast cancer similarly (Magnon et al., 2013; Hayakawa et al., 2017; Renz et al., 2018). Gliomas are deadly brain tumors caused by abnormal neuronal activity. Neuronal activity plays a crucial role in glioma progression. Growth factors released by the brain during glioma formation have an essential role. Venkatesh et al. (2019) found that synapses between neurons and gliomas depend on AMPA receptors for electrochemical communication. According to the findings, synaptic integration and electrical activity with neuronal circuits allow tumor growth. As demonstrated in diverse disease models and human tumors, Venkataramani et al. (2019) determined that neurons and gliomas can communicate directly in functioning chemical synapses between postsynaptic neurons and presynaptic gliomas. The findings suggest that neurons and glioma cells are directly synaptically connected, which may have therapeutic implications. Ameloblastoma is a potentially recurrent tumor that damages local tissue unless completely removed. Ameloblastomas are notoriously challenging to treat in the neck and head surgery field owing to the need for correct resection and restoration of the remaining defect, which can be functional and aesthetically pleasing. According to Adeel et al. (2018), between 1991 and 2009, patients with histologically proven ameloblastoma were found. The ameloblastoma was histologically proven in 15 individuals. Nine men and six women were in the group, with patients ages 20–60 years. One of our patient groups reported painless facial swelling as the main symptom. Thirteen of the patients had tumors in the mandible, and two had tumors in the maxilla.

### 2.3 Fibrous dysplasia

Fibrous dysplasia (FD) is a rare sporadic benign condition of bones in which the normal cancellous bone is replaced by fibro-osseous tissue and immature osteogenesis (Couturier et al., 2017). FTD affects both bone resorption and bone production. Adenylyl cyclase and the cyclic AMP signal pathway are activated by genetic mutations in the GNAS gene, which cause fibrous tissue and aberrant (woven) bone to form. There are several ways bone disease may be caused, including the presence of one or more bones (monostotic) or the presence of numerous bones (polyostotic). A common symptom of FD is extensive dysmorphic characteristics, bone deformity, and dental deformities in the craniofacial skeleton (Burke et al., 2017). In addition to aneurysmal bone cysts, cement-ossifying fibromas, cement-osseous dysplastic lesions, giant cell tumors, cement-ossifying fibromas, and simple bone cysts, benign fibro-osseous lesions (BFOL) of the jaws commonly occur. Other illnesses may exhibit the same symptoms; they must be distinguished. As a result of this condition, asymmetry in facial features and significant bone growth can occur. There have been reports associated with FD, especially in the orbits, of dystopia and involvement of the orbital canal. Hearing loss can result from injuries to the ossicles or external or internal auditory canals (Frisch et al., 2015). Couturier et al. (2017) followed up ten patients with craniofacial FD between 2010 and 2015. Headaches (*n* = 3), vestibular disorders (*n* = 1), and recurrent anterior uveitis (*n* = 1) were the most common complaints. Monostotic instances were all observed. Sphenoid was the most frequent bone implicated, followed by ethmoids, frontals, fronto-ethmoids, temporals, and fronto-ethmoids-sphenoids. A bisphosphonate, pamidronate, was administered intravenously to five patients: three improved within 6 months with no headaches or vestibular symptoms and two remained stable. Maxillary and mandibular alveolar bones can be damaged by craniofacial FD, resulting in occlusion problems. On a panoramic radiograph, the radiopacity may be lost due to FD of the maxillary bones (Couturier et al., 2017).

### 2.4 Human congenital disorders

Acrofacial dysostoses (Cho et al., 2016) and mandibulofacial dysostoses (MFD) relate to splicing factor gene mutations. Congenital malformations characterized by faulty pharyngeal development are MFDs, whereas limb deformities are known as AFDs (Merkuri and Fish, 2019). Cranial synostosis, a pathologic craniofacial condition, occurs when one or more cranial sutures (calvary) reattach. A healthy craniofacial structure relies on healthy sutures and unossified mesenchymal cells that form fibrous joint structures: convulsions, brain damage, intellectual disability, malformations, strabismus, and breathing and vision problems. Genetic and epigenetic changes, teratogens, and environmental factors contribute to craniosynostosis’s diverse etiology, making the syndrome extremely complex (Yilmaz et al., 2019). Osteogenesis heterotopic is a phenomenon of ectopic bone formation in soft tissues. An underlying genetic condition is less likely to trigger this condition than traumatic brain injury or surgery. Heterotopic ossification (Cohn Yakubovich et al., 2017) is a form of progressive osseous heteroplasia (POH). This form of abnormal bone growth is the opposite of FD (Ibarra and Atit, 2020). A mutation in the GNAS gene leads to progressive osseous heteroplasia, which is an autosomal dominant condition. The symptom is an ectopic intramembranous bone formation in subcutaneous and dermal tissues (Favus, 1996). In FD and POH, a somatic mutation in GNAS causes the condition in the early stages of development. Activating missense mutations develop in the somatic cells of a post-zygotic embryo (Feller et al., 2009).

### 2.5 Periodontal disease

Periodontal disease is an inflammatory condition surrounding the bone that causes irreversible damage to the tooth attachment. Smoking and hereditary vulnerability are variables that alter the host’s immunological response to microbial colonization of the periodontium, causing periodontal disease. The presence of periodontal diseases has been linked to the development of various underlying systemic illnesses (Han et al., 2014a). According to the traditional classification of periodontitis, this disorder has two kinds: aggressive and chronic forms. Moreover, periodontitis may be categorized as a chronic inflammatory disorder with multiple causes, a multicausal, complicated, chronic inflammatory condition (Loos and Van Dyke, 2020). There is a connection between periodontitis and various health problems, including cardiovascular diseases, in the general population (Hsieh et al., 2018). In addition to chronic periodontitis, the sixth most common human disease, severe periodontitis is considered one of the most frequent chronic diseases (Sanz et al., 2020). Periodontitis and gingivitis have affected patients’ quality of life, with significant adverse effects affecting function and appearance. Poor pregnancy outcomes, cardiovascular illnesses, type II diabetes mellitus (Renz et al., 2018), respiratory problems, and pneumonia in patients with hemodialysis have been linked to epidural periodontitis (Fischer et al., 2020). The global distribution of periodontal disease is a mystery, despite its strong relationship with systemic illnesses. Nazir et al. (2020) analyzed data on periodontal diseases in low-, middle-, and high-income nations, as well as adolescents and adults. The percentage of adolescents without periodontal disease was 21.2%, compared to 9.3% for adults and 9.7% for the elderly. Teenagers were more likely than adults and the elderly to experience bleeding, with 18.8% doing so after probing. A similar proportion of teenagers, adults, and elderly adults developed calculus.

### 2.6 Gingivitis

The most common form of gingivitis is plaque-induced inflammation, but other forms can significantly impact patients, including non-plaque-induced gingival disorders. There are many underlying causes of non-plaque-induced gingival lesions, but they can be pathological changes localized in the gingiva. There is a discussion on genetic disorders and developmental problems, inflammatory diseases with lesions, immunological diseases, endocrine diseases, metabolic diseases, traumatic lesions, and neoplasms (Holmstrup et al., 2018). An uncommon form of gingivitis, necrotizing ulcerative gingivitis, appears abruptly, painfully, and destructively (Dufty et al., 2017). Inflammation of the gingiva is a well-defined site-specific disease that has been extensively measured and is consistent with epidemiological studies showing its prevalence worldwide. The distinction between grading a gingival inflammatory disease at the site level and grading a gingivitis case should be evident, and a “gingivitis site” does not always imply gingivitis (Trombelli et al., 2018).

## 3 The current surgical therapy in craniofacial diseases

It is vital to clean and debride the wound entirely and remove all foreign materials before the wound can be closed. If any tissue cannot determine its viability, it should be left alone. In the following days, serial debridement should be conducted every 48 h. Corrections should then be attempted on the facial skeleton. External fixation is typically used when there is substantial bone loss with minor soft tissue injury. Internal fixation is usually used for severe bone loss with limited soft tissue injury. The issue of transplanting vascularized bone from the iliac crest or fibula to fix defects in the midface and jaw longer than 5 cm remains under discussion (Braun and Maricevich, 2017). The treatment of condylar fractures can be accomplished with preauricular, retromandibular, postauricular, or submandibular techniques. It is more often to use the preauricular and retromandibular techniques (Shakya et al., 2020). Yang et al. (2013) used a 3D simulation system to reduce the fractured portion of the mandible to its remaining segment and obtain preoperative reference data for screw size and placement. This preoperative reference was used during surgery. An assessment of the condyle using virtual reality before surgery reduces the operating time and helps achieve a better reduction and minimize operational errors. A computer-assisted preoperative simulation method called virtual surgical planning (VSP) was recently employed to treat mandibular condylar fractures (Shakya et al., 2020). Using VSP to determine the optimal screw length, position, angle, and hole size for fixing a fractured condylar section improved intraoperative efficiency (Boffano et al., 2017). A surgeon can assess and optimize various surgical alternatives by virtual assessment. Surgical reduction and stabilization are required to mend the shattered section. The correct number and placement of titanium plates and screws are required to hamper the functional stresses experienced during bone healing. The condylar neck and base are identified by two holes on either side of the fracture line on L-shaped, straight, or four-hole mini plates. In case of condylar head fracture, two screws are inserted through the lateral pole of the condyle, above the condylar neck, or below the lateral attachment of the capsule. Although titanium plates are durable and biocompatible, there remains the possibility of future failure, necessitating their removal; in this case, a re-entry procedure is needed (Kanno et al., 2018; Shakya et al., 2020). In addition to nerve and vascular damage, hearing loss, and CSF fistulas, TFB can cause serious complications. The treatments for TBF without CSF leak should be tailored individually; however, antibiotics should not be used as a preventive measure. Antibiotics should be prophylactically prescribed when there is a CSF leak, but this use is controversial. Carotid intertemporal artery damage is uncommon and involves packing the ear canal followed by carotid artery ligation or balloon occlusion of the carotid (Diaz et al., 2016). Approximately 17% of patients with TBF experience CSF leaks because meningitis is a possible consequence. A CSF fistula should be evaluated whenever fluid discharges from the ear canal or nose. Fistulas that persist for more than 7–10 days must be surgically repaired. The fistula’s location and whether brain herniation occurs through the tegmen determine the approach (Diaz et al., 2016). In treating fractures of the face, it is crucial to preserve bone, and only very little detached tissue needs to be removed first. Surgically constructed subcutaneous pouches can be used to preserve large fragments of avulsed bone for later recovery and reconstruction. A nylon suture should be placed around damaged nerves and salivary ducts (Rajguru, 2013). 3D printing can simplify reproducing a patient-specific prosthesis’s color, texture, stiffness, and form. Craniofacial prostheses can improve the appearance of the oral, nasal, and auricular areas. In addition to its use as a stopgap measure, prosthetic rehabilitation can also be used before surgical reconstruction. In most cases, CFDs are treated with prosthetics that replace the lost tissue and cover the underlying tissues. These devices are typically polydimethylsiloxane (PDMS), a flexible polymer (Nyberg et al., 2017). 3D modeling and manufacturing technologies can help in the individualized surgical reconstruction of complicated CFDs with improved tissue cutting according to preoperative plans, less time during surgery, and more cost-effective use of alloplastic and metal components (Nyberg et al., 2017). The safety and efficacy of stem cell populations was evaluated in patients with histories of craniofacial trauma or cleft palates. Eighteen individuals with cleft palates and missing teeth participated in a randomized clinical experiment in which they also had horizontal bone abnormalities. Patients could receive either autogenous block grafts or stem cell treatment. Before implant insertion, a 4-month healing interval was followed to assess the bone width for the treated locations. In summary, stem cells can be safely used for treating significant alveolar anomalies, but they cannot wholly replace large alveolar defects. Using this strategy requires more refinement to achieve the outcomes achieved with existing treatments for significant anomalies such as cleft palates (Bajestan et al., 2017). According to Kinsella et al. (2012), rat and human bone morphogenic protein-2 (rhBMP-2) is more effective than autografts in treating scarred calvarial wounds in rabbits. CT scans were performed on each animal after secondary reconstructive surgery at 0, 2, 4, and 6 weeks postoperatively. Six weeks was then allowed for all animals to be killed and histologically examined. We estimated the percentage of defects that corrected themselves over time and with therapy using a four-three mixed-model analysis of variance. A 3D-printed polymer scaffold and a signaling growth factor were used to treat a significant periodontal osseous defect in Rasperini et al. (2015). The treated area covered a 12-m distance following treatment. It has been suggested that 3D-printed image-based scaffolds may be used for periodontal reconstruction. Both materials and techniques have advanced considerably in craniofacial reconstruction during the past few years. The ideal material for these restorations is still autogenous tissue; however, the harvesting process is time-consuming and frequently results in donor site morbidity (Visscher et al., 2016). 3D-printed regeneration constructs are intended to replace deficient tissues with organic ones completely. Therefore, a construct consistently packed with cells capable of forming tissue that has also been activated for regeneration should be created. Two methods are possible: acellular printing scaffolds before implantation or printing live, cellular constructions, a technique known as “bioprinting” (Nyberg et al., 2017). Reconstructive surgery faces a whole new set of challenges in the case of abnormal CMF development due to trauma, cancer, or congenital deformities. Composite tissue allotransplantation (CTA) was developed due to recent advances in craniofacial surgery and immunotherapy (Susarla et al., 2011). Surgery, including the regeneration of alveolar bone, cement, and periodontal apparatus, is often used in surgical periodontal treatment to maximize bone volume and attachment (Wu et al., 2021). Some surgical therapies for craniofacial diseases are included in Table 1.

**TABLE 1:** Surgical therapy in craniofacial diseases.

Opinion	Materials and methods	Results	Ref.
Unilateral CLP (UCLP)	Comparing cephalometric values of untreated cleft and non-cleft children from prior growth studies can identify differences in growth patterns. Examine 8-year-old children with UCLP before and after orthodontic treatment and alveolar bone grafting	Researchers found that standards should be used for young UCLPs before orthodontic treatment, which may be helpful for future treatment planning by clinicians	Küseler et al. (2021)
AR	A surgical procedure for craniofacial FD recontouring is described using the authors’ AR navigation system	This new technology may improve the safety and effectiveness of craniofacial FD recontouring surgeries	Liu et al. (2021)
AFT	This procedure is evaluated in cases of traumatic and postsurgical craniofacial abnormalities. Its minimally invasive nature can lower risk and provide better outcomes than current reconstructive options	Compared with traditional reconstructive treatments for craniofacial abnormalities, AFT is safer. After 3 months, AFT achieves long-term volume stability and is efficient, consistent, and reliable. The treatment has changed the lives of many patients based on their reports	Bourne et al. (2021)
Dental implant	Study participants with periodontitis and dental implants with or without bone grafts were compared to those with vitamin C supplementation on wound healing	Vitamin C supplementation could improve postoperative healing after dental implant surgery in patients with chronic periodontitis and those receiving Bio-Oss collagen grafts or GBR. However, vitamin C supplements did not affect easing the discomfort after surgery	Li et al. (2018)
Static computer-assisted implant surgery (CAIS)	Freehand implant surgery and static CAIS were compared in a single edentulous space about the accuracy of implant placement	The static CAIS technique gave better implant placement accuracy in a single-educated area than freehand placement	Smitkarn et al. (2019)
Posterior pharyngeal fat grafting	Studying the factors affecting speech outcome after posterior pharyngeal fat grafting to treat velopharyngeal insufficiency (VPI)	Individuals with VPI can improve their speech function with posterior pharyngeal fat grafting. However, the size of the gap, the number of previous palate surgeries, and the referral patterns influence the outcome	Denadai et al. (2020)
Contracted endodontic cavity	Using nickel–titanium rotary tools to shape endodontic cavities with confined endodontic cavities	TECs preserve the canal morphology better than CECs during shaping, especially at the apical level	Alovisi et al. (2018)
Dental RECUR	A study to test the effectiveness of delivering the DR-BNI oral health intervention by dental nurses in preventing the reoccurrence of dental caries in children whose primary teeth have been removed	This approach, as an inexpensive and low-intensity intervention, could significantly decrease the rate of dental caries recurrence in children	Pine et al. (2020)
A-PRF	A-PRF alone or in combination with FDBA is tested for its efficacy in improving ARP and alveolar dimensional stability	According to this study, a-PRF is effective for ridge preservation alone or in combination with FDBA. A-PRF with and without FDBA is compared here with only FDBA in the first randomized controlled trial of ridge preservation	Clark et al. (2018)
ARP	Compared with unaided socket healing, ARP was more effective. Additional goals included determining the influence of local phenotypic characteristics on the volumetric decrease of the alveolar bone	The assessment of reported postoperative pain was the same across groups at each follow-up visit. Although both treatment groups had substantial alveolar ridge remodeling, ARP was superior to EXT because it was more effective in maintaining alveolar bone and, as a result, was less likely to require additional bone augmentation in the future	Avila-Ortiz et al. (2020)
Virtual surgery	Guided virtual surgery was compared to traditional surgery regarding the angular deviation of single dental implants	Based on the data, the angle difference between the clinical implant location and the virtual was slightly smaller than when stereolithographic guidance was used instead of conventional guides	Magrin et al. (2020)
Excess intraoperative fluid administration	In children undergoing dental surgery, excess intraoperative fluid administration can be determined either by an automatic pump-delivery system or manually by a gravity-drip device	Utilizing an automated pump-delivery system for intraoperative fluid administration reduced excessive intravenous fluid administration in dental surgery patients	Bowes et al. (2020)
Amelogenin–chitosan hydrogel	The scientists reported a novel protocol to reconstruct the superficial enamel using a newly developed hydrogel	*In situ* regeneration of apatite crystals may create thick enamel-restoration interfaces, whereas the enamel-like structure governed by amelogenin assemblies may enhance the properties of etched enamel. In addition, chitosan hydrogel is simple to use and may prevent dental caries by preventing bacterial infections	Ruan and Moradian-Oldak (2014)
HA gel at 45 days on the microbiome of implants	It has been 1 year or more since the peri-implantitis started. Randomized controlled studies have been performed on peri-implantitis patients. After 45 days of treatment, another swab was taken containing the samples. The methods that sequence 16S rRNA were used for assessing the effect of HA gel on the subgingival microbiota	In the early stages of the disease, HA could control and decrease the relative number of microorganisms that caused peri-implantitis, particularly early colonization bacteria. No effect of HA was seen on non-oral genera. By applying HA to advanced stages of peri-implantitis, bacteria diversity was reduced, suggesting a barrier to bacterial colonization at the peri-implant site	Soriano-Lerma et al. (2020)
Xenogeneic block loaded with rhBMP-2	Two hundred and four patients (control and test) underwent primary augmentation using either rhBMP-2-loaded xenogeneic or autogenous blocks. After augmentation surgery and 4 months before implant insertion, CBCT scans were used to measure the ridge width (RW). A surface scan was taken before augmentation and 4 months later to conduct profilometric analysis	A comparable amount of ridge widening was achieved with both treatment methods. For the healing process, neither group shrank more than the control group. Also, the shape of soft tissues was little affected by hard tissue augmentation	Thoma et al. (2019)
Analyzing the angiogenic potential. A procedure for autogenous bone augmentation of the maxilla	The control group received autogenous bone with graft materials, including hydroxyapatite, *Ricinus communis* polymer, and demineralized freeze-dried bone allograft. Autogenous bone was used solely in the control group	Although different graft materials have effectively augmented the sinus floor, much remains unknown about interconnected bone growth and angiogenesis	Boëck-Neto et al. (2009)
Submental intubation	Intubating submental fractures, complicated fractures of the maxilon or mandibular jaws involving facial bones, and nose-orbit-ethmoid (NOE) fractures	Panfacial, nose-mouth-eye, and craniofacial fractures are successfully treated safely with submental intubation since tracheostomies are avoided. The IMF is not affected by it throughout the intraoperative period	Mishra et al. (2020)
Modified Keen technique	In the case of surgical excision of coronoid fractures, the mandibular ramus of the mandible is usually exposed through an intraoral posterior incision. An alternative and innovative method for the coronoid process was developed based on the Keen approach to the lateral midface	It employs an anterior intraoral incision that can be easily retractable and sealed, allowing direct visualization of the coronoid process without an endoscope. A concurrent midfacial fracture can be stabilized with the method. Therefore, the modified Keen method for coronoidectomy is preferable for treating trismus using an intraoral approach	Roy and Reid (2021)
Titanium mini-plate systems	The interleukin 1 (IL-1) and IL-6 concentrations were measured in macrophages derived from THP-1 monocytes after using 3D titanium mini-plate systems and customized screws in the surgical treatment of condylar fractions	Despite significant effects on osteoclast activity and bone remodeling processes after implantation, titanium plates are a safe and neutral material for humans	Sikora et al. (2020)
ATTM	Structural optimization was performed on optimally designed ATTM plates created through additive manufacturing and anatomical thin titanium mesh (ATTM) plates	Their results indicated that the ATTM plates with sufficient strength to withstand bending were best designed using FE and Taguchi analyses. Optimally designed ATTM plates with patient-matched face shapes manufactured by AM provide excellent support for ZMC comminuted broken bones	Wang et al. (2018b)
Microinjector	The microinjector was developed to provide bone graft replacement for periodontal disease bone regeneration. Instead of using the GBR system, this gadget would be used instead	Based on these results, the innovative microinjector may be suitable for dental applications	Tsai et al. (2016)
Biomimetic multilayered palate substitute	A highly biomimetic multilayered palate replacement with oral mucosa tissues and bone was created with rabbit cells and biomaterials exposed to nanotechnology based on plastic compression	In the present study, tissues can partially differentiate after *in vivo* grafting, indicating the possibility of creating a full-thickness multilayered palate prosthesis	Martín-Piedra et al. (2017)
Non-hairy punch graft	Hair follicles were confirmed to be involved in healing venous leg ulcers after transplanting hair follicle-containing punch grafts and non-hairy punch grafts	Punch grafts obtained from non-hairy sites perform better than scalp punch grafts for autologous transplantation of terminal hair follicles. Effective surgical treatment for chronic venous leg ulcers is hair punch grafting	Martínez et al. (2016)
CMX	To determine a difference in smile aesthetics with or without a CAF as a root covering surgery	Compared to how the SEI was achieved by CAF alone, the SEI achieved by CAF + CMX did not differ from what was achieved by CAF alone 12 months later	Rotundo et al. (2021)
Socket shield technique	Comparison of implant stability and pink aesthetic score evaluation between the immediate implant and the socket shield technique placement and immediate temporization and vertical changes in buccal cortical bone	In combination with immediate temporization, socket shielding prevents the loss of labial bone after tooth extraction. To determine whether grafting the jumping gaps will affect bone loss reduction, more research is needed	Abd-Elrahman et al. (2020)
Photobiomodulation	The removal of mandibular third molars is often associated with discomfort, trismus, and swelling of the face after the procedure. Photobiomodulation was used in this study to determine if it may reduce postoperative adverse effects following the removal of mandibular third molars by surgery	This research suggests that photobiomodulation may reduce swelling and pain after mandibular third molar surgery. This is a successful alternative for the surgery alone, and it enhances patients’ quality of life	Singh et al. (2019)
Microneedling	The effectiveness of microneedling combined with PRP or TCA peeling for the face is being evaluated and compared	According to histometrical and histochemical evaluations, most volunteers improved clinically after treatment; however, dermarolling in combination with PRP provided the best results	El-Domyati et al. (2018)

## 4 Regenerative medicine in craniofacial diseases

One of RM’s goals is to treat damaged tissues or organs. The complexity of the human body poses considerable challenges, even though considerable progress has been made lately (Atala and Forgacs, 2019). Our body’s tissues, organs, and organ systems are built on stem cells, which are at the center of the RM strategy. Four types of stem cells exist: omnipotent, multipotent, pluripotent, and totipotent. There is only one totipotent stem cell in humans, the Zygote, which forms the basis of an entire organism. According to the regenerative characteristics of stem cells, they are classified as embryonic stem cells (ESCs), tissue-specific progenitor stem cells (TSPSCs), mesenchymal stem cells (MSCs), umbilical cord stem cells (UCSCs), bone marrow stem cells (BMSCs), and induced pluripotent stem cells (iPSCs) (Bijukumar et al., 2018). The RM combines molecular and cellular bases, bioengineering, and material sciences to restore the function of the organ/tissue (Borrelli et al., 2020). Restoring the natural facial appearance with custom face prostheses is called prosthetic rehabilitation. Various plastic surgery procedures can be used to reconstruct the craniofacial region, including fixation devices, cutting guides, implanted medical equipment, and practice models. Regeneration creates a new craniofacial tissue through stem cells and biologically active scaffolds (Nyberg et al., 2017). One of the most challenging surgeries is the secondary reconstruction of facial abnormalities following posttraumatic injury. Primary reconstruction may not always be possible unless the comminution or surgical treatment is severe (Aman et al., 2019). Several surgeons are choosing internal fixation and open reduction for repairing condylar fractures due to new technologies, improved fracture management, and evidence of superior results in the literature. Due to the difficulty of surgically treating such fractures, many factors must be considered to ensure a satisfactory outcome (Shakya et al., 2020).

### 4.1 Stem cell therapy in craniofacial diseases

Oral stem cells can effectively replace BMSCs with high bone production potential. Stem cells from apical papilla (SCAP) and stem cells from human exfoliated deciduous teeth (Jahanbin et al., 2016) require human testing in clinical trials before their use (Sybil et al., 2020). The risk of facial paralysis following a TBF is approximately 6%–7%; 25% experience immediate total paralysis, whereas 75% experience partial or incomplete paralysis. When the facial nerve has been injured, patients with an acute-onset, complete facial paralysis should be assessed between 3 and 7 days later, allowing time for Wallerian degeneration. Depending on the severity of the damage, clinical assessment could be performed using facial nerve stimulation using the Hilger stimulator, electromyography (EMG), or electroneurophysiology (ENOG). Patients with refractory traumatic facial nerve paralysis can use neurons and MSCs. Cell-based Schwann cell synthesis *in vitro* seems possible *in vitro*, especially when paired with material bridges, as animal studies have investigated neural and ADSCs (Diaz et al., 2016). Research is currently being conducted to use autologous stem cells to treat CFDs. Patel et al. (2013) reported that CSPCs can create elastic and long-lasting tissue regeneration. It is a connective tissue that makes up the pulp of the tooth. This tissue is made up of different kinds of cells, and it contains walls that protect the integrity of the tissue. In 2000, Griffos et al. could isolate some stem cells from impacted third molars as a new source of stem cells (Maxim et al., 2015; Estrela et al., 2011). Cells known as DPSCs continually proliferate and differentiate in their culture medium, depending on what medium they have been placed in. In some animal models, the ability of these cells to proliferate and differentiate into odontoblast-like cells, bone-like tissue, and pulp-like tissue shows their potential for use in cell-based therapies (Han et al., 2014b). Another resource is the apical papilla. SCAPs produce odontoblast-like cells *in vivo* and are ideal for regenerating odontogenic tissue *in vitro*, producing dentin *in vivo*, and developing roots (Hemmat et al., 2010).

**FIGURE 1:**
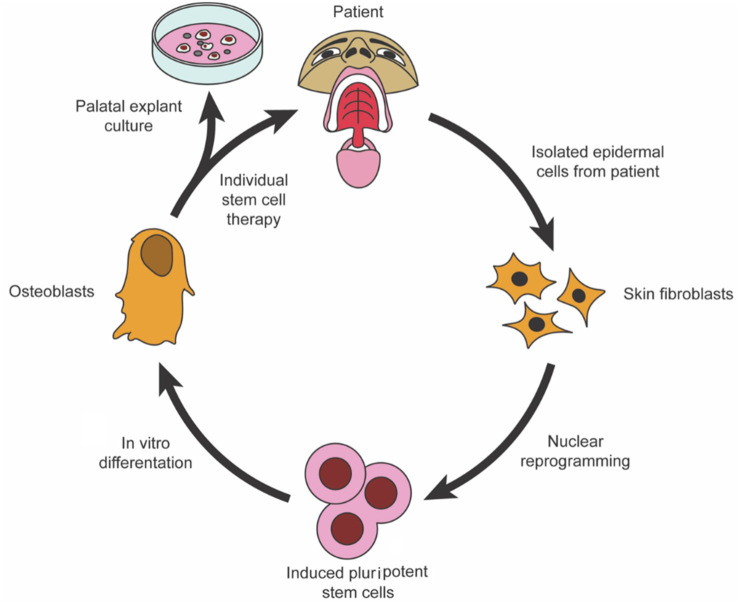
To restore palatal bone, iPSCs were isolated and programmed (Oliver et al., 2020).

Using periodontal stem cells in patients with periodontal deficiencies has been proven safe without immunological or inflammatory adverse effects. Consequently, successful periodontal tissue regeneration requires the recruitment of locally produced progenitor cells, resulting in differentiation into bone-forming cells, cementum, and PDL (Liu et al., 2019). Wang et al. (2016a) created a technique for injecting stem cells into the body using encapsulated MSC derived from iPSC. The microbeads remained viable even after injection. The microbeads released cells with a 10-fold increase in live cell density from 1 to 14-day. In the cells, osteogenic markers were upregulated along with mineral deposition. Therefore, they concluded that CPC-microbead-iPSMSC might benefit bone regeneration in orthopedics, dentistry, and craniofacial applications. When polymers break down, they create porosity, which can improve the degradation of weakly degradable injectable CPCs. The long-term biological performance of CPC-PLGA has not been determined, although it is biodegradable. Injectability might be improved by adding carboxymethyl cellulose (CMC). The *in vivo* results of a long-term study on CPC-PLGA without/with the lubricant CMC were compared with those of a devitalized bovine bone mineral predicate device (DBBM), Bio-Oss^®^. A 26-week study found that CPC-PLGA was helpful in bone regeneration, with >40% of new bone growing (Grosfeld et al., 2016). As part of their study on the feasibility of regenerating periodontal abnormalities with allogeneic BMMSCs in rats with periodontitis, Du et al. (2014) used rat models of periodontitis. Invasions of BMMSCs were performed on rats extracted and mixed with a 0.9% sodium chloride solution. Those in the control groups received NaCl solution at 0.9% or no treatment. According to the study, local injections of BMMSCs may act as an anti-inflammatory and immune modulator in periodontitis-related abnormalities treatment. For peri-implantitis defect regeneration, Park et al. (2015) used canine periodontal ligament stem cells (PDLSCs) *in vivo* in a dog. The PDLSCs of dogs were transduced using adenoviral vectors expressing BMP2. Six beagle dogs with peri-implantitis caused by ligature implantation were treated with HA particles and collagen gel containing autologous PDLSCs or BMP2/PDLSCs. The drug was delivered through PDLSCs to promote re-osseointegration and new bone formation. There is a schematic of palatal regeneration using stem cells in Figure 1.

### 4.2 Growth factor therapy in craniofacial diseases

In addition to vascularizing the bone and providing osteoprogenitor cells, the periosteum also contributes to bone growth and repair. Using PRP/BMSC gel membranes for regenerative bone repair, El Backly et al. (2013) presented a periosteal replacement wrapped around an osteoconductive scaffold. By adjusting the composition of PRP/BMSC gel membranes, it controlled the release of platelet-derived growth factor-β (PDGF-β) and VEGF. Lastly, in a mouse model, the PRP/BMSC gel membrane periosteal substitute was evaluated *in vivo* for its ability to biomimic a periosteal response enhancing bone regeneration. Yamada et al. (2013) also examined whether injectable regenerative bone pills containing PRP and stem cells could help alveolar deficits repair the functional bone. With no negative side effects, all patients experienced a significant increase in bone volume. At 3 months, newly generated bone regions reached levels comparable to natural bone, a significant improvement over the preoperative level. A significant amount of bone resorption did not occur during a long-term follow-up. Patients with masticatory impairments were helped by injectable tissue-engineered bone. Using bone tissue engineering for treating patients is a unique but effective treatment method. How each signaling pathway affects the overall shape of the anterior zebrafish neurocranium was determined, as well as how it affects the formation of the ethmoid plate in Cusack et al. (2017). Researchers determined that different signaling routes affect the ethmoid plates’ shape, size, and shape in dose- and time-dependent ways. Shen et al. (2021) hypothesized that VEGF or nerve growth factor (NGF) therapy during SCAP development might alter the expression of the osteogenic and endogenic markers. Using VEGF/NGF in regenerative endodontic procedures can reveal dentinogenic/neuronal/healing markers in SCAPs. Recent studies on growth factors and stem cell therapy in craniofacial and dental trauma and disease are summarized in Table 2.

**TABLE 2:** Stem cell and growth factor therapy in craniofacial diseases.

Opinion	Materials and methods	Results	Ref.
Rat MSCs (rMSCs)	Stem-cell-cultured conditioned medium is being investigated for its impact on bone regeneration. The MSC-cultured conditioned medium increased osteogenic marker gene expression, proliferation, and migration	By mobilizing endogenous stem cells, MSC-CM appears to be able to restore bone. Stem-cell-cultured conditioned media are a novel approach to bone regeneration that take advantage of the paracrine functions of stem cells without having to use transplanted cells	Osugi et al. (2012)
iPSCs	*In vivo* bone regeneration from transplanted iPSCs was examined	Biomaterials containing nanofiber scaffolds and iPSCs may be able to regenerate bone in critical-size defects	Hayashi et al. (2016)
hBM-MSCs	Comparing marrow-derived and amniotic fluid-derived human MSCs for their osteogenic differentiation and regeneration capabilities	Compared to BM-MSCs, AF-MSCs can repair bone more effectively *in vivo*. Hence, AF-derived MSCs are suggested as an alternative to BM-MSCs in bone regeneration surgery and spinal fusion. Additionally, gel foam was shown to function as a biocompatible, biodegradable, osteoinductive scaffold *in vivo*	Mossaad et al. (2019)
Autologous BMMNCs	Alveolar cleft repair may be improved using autologous BMMNCs and nanohydroxyapatite combined with PRF	Combining PRF, nanohydroxyapatite, and BMMNCs improves bone healing in alveolar cleft defects, replacing alveolar bone transplantation to treat clefts	Al-Ahmady et al. (2018)
Adult MSCs and FGF-2	Similarly, MSCs and FGF-2 appear to be equally effective in promoting bone regrowth in craniofacial bone repair	FGF-2 priming plays a crucial role in tissue engineering for cranial bone repair	Novais et al. (2019)
Human DPSCs and amniotic fluid stem cells (hAFSCs)	The repair of critical-size lesions of the cerebral spine was carried out using human stem cells like AFSCs and DPSCs in immunocompromised rats	Our study suggests that bioengineered stem cells/fibroin structures may prove to be a feasible procedure for treating severe skeletal deformities in humans through the restoration of craniofacial structures	Riccio et al. (2012)
ADSCs	The ADSCs were harvested without cell culture, demonstrating the ability to alleviate the harmful effects of radiation on fracture healing with this unique harvest and distribution strategy	ADSCs promote irradiation fracture healing without the need for cell multiplication in culture in the absence of minimally processed non-cultured stem cells. In addition to further refining the cell collection and delivery methodologies reported in this work, increased union rate and bone quality can be achieved with minimally processed, non-cultured ADSCs	Lynn et al. (2020)
BMSPC	Cells were sown directly onto the lesion using autologous stem and progenitor cells taken from bone marrow	Six months after implant placement, a dental prosthesis was inserted to complete the patient’s functional and cosmetic recovery	Rajan et al. (2014)
TRCs	CD90^−^ and CD14-positive cells are significant components of tissue repair cells (TRCs) derived from bone marrow. TRC cells were used to repair localized abnormalities of the skull and face	In treating alveolar bone abnormalities, TRC transplantation appears safe and can speed up the bone repair process, allowing for the restoration of oral implants. The findings of this study suggest that TRC therapy should be investigated further for treating craniofacial abnormalities	Kaigler et al. (2013)
Periodontal ligament stem/progenitor cells (PDLSCs)	This study aims to produce integrated cementum on the root surfaces of human teeth PDLSCs using growth factor-releasing scaffolds and periodontal ligament stem/progenitor cells	The results are significant because stem cells can be used to regenerate cementum, which is an essential aspect of implant-based regeneration	Cho et al. (2016)
hPDLSC	To control PDL and cementum regeneration (rhBMP-2), the authors used the sheet engineering method with human PDL stem cells (hPDLSC). hPDLSCs isolated from extracted human teeth were treated with rhBMP-2 and grafted onto synthetic micro/macroporous biphasic calcium phosphate (MBCP) blocks to induce osteogenic differentiation	Using the rhBMP-2-hPDLSC sheet method, one may be able to simultaneously regenerate mineralized tissue and fibrous tissue at the periodontal complex	Park et al. (2020a)
BMSCs	This study investigated the combined use of dentin matrix protein-1 gene-modified BMSCs and Bio-Oss^®^ for maxillary sinus floor augmentation (MSFA) *in vivo* in an animal model	A supplement to Bio-Oss^®^ may be administered to MSFA dogs that contain BMSCs with the DMP1 gene to improve new bone formation and osseointegration of dental implants	Liu et al. (2016)
hiPSCs	Through seeding on biofunctionalized macroporous CPCs, researchers have compared the regeneration of bone in rats with hUCMSCs, hBMSCs, and hiPSCs	Biofunctionalized macroporous CPC-stem cell constructs were more effective in assisting bone regeneration than cell-free CPCs; hiPSC-MSCs and hUCMSCs were viable alternatives to hBMSCs, and hiPSC-MSCs compared to hBMSCs	Wang et al. (2015)
ADSCs	ADSCs have shown promising agents in periodontal tissue regeneration	SRP can be treated effectively with ADSC, including their Exo	Mohammed et al. (2018)
MSCs	Researchers compared bone regeneration *via* autologous bone transplantation *versus* bone marrow-derived MSCs	MSCs were found to cause significant bone growth in this large-scale clinical trial in humans, with no significant negative side effects. This innovative method can be an alternative to the current gold standard because it is an innovative addition approach that deserves further exploration	Gjerde et al. (2018)
Stem cells	MSCs were found to cause significant bone growth in this large-scale clinical trial in humans, with no significant negative side effects. This innovative method can be an alternative to the current gold standard because it is an innovative addition approach that deserves further exploration	The researchers found that these stem cells possess innate abilities to replace damaged skeletons in cell-based therapies, revealing the complexity of the congenital disease and regenerative medicine by analyzing their true identity as skeletal stem cells	Maruyama et al. (2016)
ACO	ACO technology based on scaffolds is effective on both a technical and biological level	Young individuals with cleft alveolar bone deformities can undergo less invasive therapy with fewer health complications. More clinical trials and *in vivo* tests are necessary to establish the benefits of this novel therapy	Berger et al. (2015)
Graft	Their study compared the biological characteristics of transplanted bone using the two methods of graft revascularization and bone remodeling	Each procedure was successful in implant implantation and prosthetic rehabilitation based on the findings. Both procedures led to revascularization and reshaping of bone tissue. Their histological findings were markedly different. The block graft outperforms particle grafts related to implant-to-bone contact and bone fill values; however, the donor site morbidity must be addressed	Rocchietta et al. (2016)
MSC	By analyzing their cellular characteristics and surface marker profiles by flow cytometry, these potential progenitor cells were compared with MSCs and nasal septum chondrocytes	The migratory subpopulation of cells can be used *in vivo* for cartilage regeneration because they tend to migrate. Using unseeded biomaterials to mobilize local progenitor cells for cartilage regeneration is ideal for *in situ* tissue engineering based on the *in vivo* mobilization of nasal cartilage progenitor cells	Elsaesser et al. (2016)
ADSC	ADSC sheets were produced from the epididymal adipose fat of rats, and it was used in wound healing	As a result, this therapy might constitute the first step toward developing more effective wound-healing treatments for diabetics	Kato et al. (2017)
AV loop model	The AV loop paradigm allows for soft tissue healing that has a unique microvascular structure	When used with the AV loop approach, collagen-based scaffolds can be useful in soft tissue regeneration. As tissue-engineered free flaps might serve as a customized therapy idea for critical wounds in the future, these scaffolds show various kinds of proliferation, cell migration, and angiogenesis	Schmidt et al. (2017)

### 4.3 Current biomaterial therapy in craniofacial diseases

**FIGURE 2:**
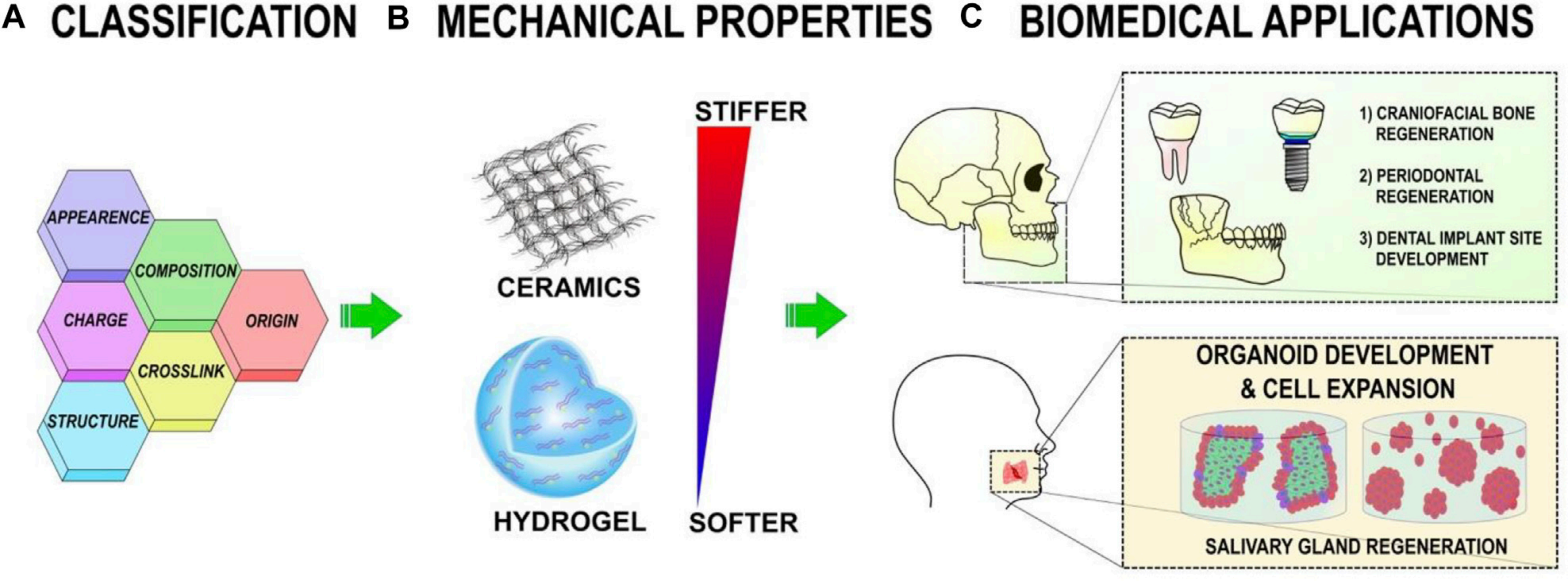
**(A)** Polymeric scaffolds can be categorized based on their content, structure, appearance, origin, cross-linking, and charge. **(B)** The mechanical properties of polymeric scaffolds can be altered to affect cellular activity. **(C)** Polymeric scaffolds help regenerate the oral, dental, and craniofacial regions in tissue engineering (Wu et al., 2021).

Clinically effective bone repair has been enabled by developing biomaterials that are viable alternatives to autologous and allogeneic grafting methods. A biocompatible scaffold is often incorporated into surgery to induce new bone formation by allowing cells to migrate, multiply, and differentiate. Numerous biomaterials have been used in craniofacial bone augmentation. Inorganic and organic materials are often separated. CaP bioceramics are a common type of inorganic scaffold, whereas organic scaffolds are made from natural or synthetic polymers (Thrivikraman et al., 2017). The purpose of biomaterials is to replace parts of living systems or provide similar functions. Optimal mechanical strength, osteoconductivity, and cell adhesion and proliferation are essential characteristics in biomaterials that serve as tissues (Tevlin et al., 2014). Congenital disorders,trauma, and bone recessions are craniofacial bone abnormalities. Tissue engineering is entered to help bone regeneration, allowing patients to benefit from the supporting effects of 3D materials combined with the synergistic effects of osteoinductive chemicals and recruited stem cells (Martín-Del-Campo et al., 2019). Figure 2 presents an overview of the polymeric scaffold characteristics and categorization.

#### 4.3.1 Bioactive materials therapy in craniofacial diseases

Providing bioactive polypeptide growth factors directly to the root surface can promote periodontal regeneration. This promotes wound healing, which results in new cementum and connective tissues. PDGF and IGF-I, for example, are effective in regenerating periodontal tissues in animals such as beagle dogs and monkeys (Han et al., 2014a). A study on n-HA/PA composite scaffolds with BMP-7 transduced MSCs in mandibular defect healing has been reported by Li et al. (2010). Radiographic, biomechanical, histomorphometric, and histological analyses of their mandibles were conducted. Group A animals developed more bone and mineralized than group B animals 4 and 8 weeks after implantation. Group B animals had more than group C. However, no differences were found at 16 weeks after implantation. Researchers found that MSCs-n-HA/PA composites transduced with BMP-7 dramatically accelerated bone formation. Using n-HA/PA scaffolds in combination with BMP-7-mediated *ex vivo* gene transfer may be another way to treat mandibular abnormalities (Li et al., 2010). Biomimetic synthesis can be used to restore and prevent damaged enamel. In recent years, it has been proven that chitosan–amelogenin (CS-AMEL) and calcium phosphate (CP) can be used to form an enamel-like layer that adheres to natural teeth surfaces seamlessly (Mukherjee et al., 2016). Prajapati et al. (2016) identified a unique function of the matrix metalloproteinase-20 (MMP-20) to prevent the entrapment of undesired organic materials in developing enamel crystals by cleaving precise amelogenin. Hydroxyapatite crystal crystallizes when MMP-20 regulates their growth morphology. In another study, MMP-20 was used to produce artificial enamel through a biomimetic process. Compared to the original enamel, both modulus and hardness were significantly enhanced by MMP-20CS-AMEL hydrogel. The findings of this study are significantly closer to achieving biomimetic enamel renewal, even though they require further study to include other enamel matrix proteins (Prajapati et al., 2018). Using hydroxyapatite, calcium sulfate hemihydrate, and Haladen collagenase (HAP/CS/HA-Col), Subramaniam et al. (2016) developed an alveolar bone replacement using hydroxyapatite, calcium sulfate, and Haladen collagenase. A WST-1 assay and mechanical testing evaluated the composite material’s biocompatibility. By using micro-CT images and histological testing, HAP/CS/HA-Col composites were validated by *in vivo* bone production in rats with alveolar bone deficiencies. Results showed that collagenase-infused HAP/CS/HA composites can effectively regenerate alveolar bone and that the same method may be applied to other tissues damaged by injury or disease. Extensive bone grafting remains a sensitive procedure due to the long and challenging integration of the grafted material into the physiological architecture. According to Simonpieri et al. (2009), platelet concentrates may speed up the integration process by speeding up the healing of mucosa and bone. Additionally, PRF membranes are functional during challenging implant rehabilitations, promoting periosteum healing and maturation. An extensive healing process on a PRF membrane layer is connected to the thick peri-implant gingiva, which may be responsible for the reduced marginal bone loss. Microthreaded collars and platform switching improved this result even more. A novel approach for improving the ultimate aesthetic outcome involves multiple healing on PRF membranes.

#### 4.3.2 Polymer, graft, and composite therapy in craniofacial diseases

These scaffolds are highly promising for tissue regeneration because of their many advantages. An array of biomaterials has been proposed for injectable scaffolds. A biomaterial may be classified according to its natural or synthetic source. In addition to being produced from natural resources, natural biomaterials exhibit biological recognition, which may aid in the adhesion and development of cells. They usually do not cause inflammatory or immunological reactions, and they are biocompatible and biodegradable. Conversely, natural materials present potential health risks, such as introducing diseases from batch to batch. Synthetic biomaterials are created as alternatives to natively generated ones to address these issues. Massive-scale production of biomaterials with well-controlled properties can be implemented. Synthetic biomaterials do not transmit pathogens (Chang et al., 2017). Among these biomaterials are polymers. A polymer is a chemical compound with long chains of atoms linked together by covalent bonds. Collagen, for example, can be synthesized or can be made naturally (Koons et al., 2020). The temporomandibular joint (TMJ) tissues can be repaired using tissue engineering techniques that use biodegradable polymeric scaffolds. These sponges may provide a platform for cell infiltration and remodeling. These sponges include synthetic polymer poly(glycerol sebacate) scaffolds and natural gelatin scaffolds. The authors examined the regeneration capability of these scaffolds combined with magnesium (Mg) (MCC) using a new fibrocartilage defect model in goat mandibular condylar cartilage. An osteochondral lesion was produced bilaterally in a mandibular condyle in adult Spanish Boer goats. A 1 mm diameter drill hole was made on the articular surface to create a trough defect. Tests included an empty control without an implant, a PGS matrix with magnesium ions, a gelatin matrix with magnesium ions, and a gelatin matrix with magnesium ions and trimagnesium phosphate (TMP) powder. A 3-month recuperation period was given to the goats before tissue samples were taken (Chin et al., 2018). In craniofacial microsomia, the skull and face are asymmetrically developed before birth. Developing resorbable scaffolds is an alternative to help restore muscle function and improve symmetry in the face while reducing risks to the donor site. To create two resorbable weft-knitted scaffolds (NPRs), Deshpande et al. (2020) used Poly (ε-caprolactone) multifilament yarns with auxetic architecture that had a negative Poisson’s ratio. Two knitted textile scaffolds (called PCL A and PCL B) utilize multifilament yarns made from poly(-caprolactone). Both samples reached porosities of more than 90% using an open-weft knit construction. Although PCL A and PCL B did not unravel, PCL A had better dimensional stability than PCL B. Following 7 days in cell culture, PCL fabric A promoted cell development more effectively than PCL B (Deshpande et al., 2020). Schleund et al. (2019) demonstrated a novel method to repair extensive mandibular discontinuities with autologous iliac bone marrow allograft seeded with radial forearm flap vascularized with autologous iliac bone marrow aspirate. In addition to the iliac crest’s low morbidity rate, hematopoietic stem cells and MSCs may develop into osteoblasts when seeded on an allograft scaffold. BTE is now often achieved using bioceramics, but bone remains the ideal substrate for engineering bone, and only allografts prevent donor-site morbidity. In BTE, one of the most challenging aspects is vascularization, which can be achieved, for example, by using pedicles or free flaps. The radial forearm flap was chosen considering the length of the pedicle and the authors’ need for a little soft tissue (Schlund et al., 2019). Zaky et al. (2017) examined the mechanical properties of poly(glycerol sebacate) (PGS), compatibility with osteoprogenitor cells, and application to a rabbit ulna considerable defect regeneration *in vitro* and *in vivo*. Polylactic acid/glycolic acid polymeric scaffolds were compared *in vitro* to a stiffer PGS with similar porosity and interconnectivity. According to Zaky’s research, because PGS is more closely related to osteoid tissue than mineralized bone, it promotes skeletal maturation by allowing osteoprogenitor/stem cells to differentiate on a substrate that is mechanically closer to how mature bone forms (Zaky et al., 2017). Oncologic head and neck reconstruction using autologous fat grafting was evaluated by Vitagliano et al. (2018) for surgical, oncological, and aesthetic outcomes. Based on their analysis of the data from this study, they discovered that autologous fat grafting might complement or replace reconstructive flaps in oncological head and neck reconstruction, with fewer complications and no evidence of cancer recurrence (Vitagliano et al., 2016).

#### 4.3.3 Collagen therapy in craniofacial diseases

Natural biomaterials, such as collagen, are widely used in injectable form. Dentin, bone, and PDL are the leading organic components of many mammals (Chang et al., 2017). Due to the complex and dynamic features of native/natural tissues, RM strategies aimed at retreating articular cartilage have a considerable challenge ahead. Parmar et al. (2015) modified streptococcal collagen-like two proteins with chondroitin sulfate (CS)-binding peptides, then cross-linked with an MMP7-responsive peptide to form biodegradable hydrogels. It was discovered that this biomaterial has the potential to influence cellular processes and build viable tissue-engineered structures for RM. Injuries to the skull and face may be treated with these materials. Primary cilia are needed for bone formation *via* intraflagellar transfer (IFT). According to Yamaguchi et al. (2020), IFT20, the minor IFT protein in the IFT-B complex, plays a crucial role in collagen synthesis in mice.

The deletion of Ift20 in craniofacial osteoblasts caused facial bone defects. By deleting Ift20, collagen protein levels remain unchanged, but collagen cross-linking is severely altered. Research indicated that IFT20 regulates telopeptidyl lysine hydroxylation and cross-linking in bone, a critical step in collagen synthesis. The success of regenerating tissues and bones in the oral and craniofacial regions can be attributed to biodegradable synthetic scaffolds. Qian et al. (2019) created an electrospun poly-lactic-co-glycolic, acid/polycaprolactone, and silver-coated collagen scaffold (PP-pDA-Ag-COL) to improve osteogenic and antibacterial features. A PP-pDA-Ag-COL scaffold inhibited the pathogenesis of mouse periodontal disease (31.8%) and promoted alveolar bone regeneration. They have demonstrated improved biocompatibility and osteogenic and antibacterial properties of our unique PP-pDA-Ag-COL scaffold for alveolar/craniofacial complex tissue reconstruction. Using collagen scaffolds and human ASDCs for oral mucosal and calvarial bone regeneration, Wang et al. (2018) utilized resveratrol (RSV), affecting MSC differentiation. A collagen scaffold containing RSV (/RSV) scaffold was assessed *in vivo* and *in vitro* for wound healing and bone reconstruction. On a collagen scaffold, RSV induced a more significant differentiation of hASC, indicating that scaffolds containing collagen and RSV can stimulate the growth of craniofacial tissue (Wang et al., 2018a). A group of mineralized collagen scaffolds was developed for bones, and complex tissue reconstruction using CMF was altered with zinc ions in Tiffany et al.’s (2019) study. Zinc sulfate is added to mineralized collagen-glycosaminoglycan precursor suspensions and freeze-dried to form porous biomaterials that are zinc functionalized. In addition to promoting zinc transporter expression, zinc functionalized scaffolds promote mineral biosynthesis, osteogenic differentiation, and stem cell viability.

#### 4.3.4 Gelatin therapy in craniofacial diseases

Restoring function to periodontal tissues includes root bio-modification, guided tissue regeneration, bone replacement grafting, soft tissue grafting, and a combination of all procedures (Nyamsuren et al., 2018). A variety of bone transplantations have been tested for their ability to induce new bone growth. A list includes alloplastic materials (generally synthetic fillers), autografts (same individual tissue transferred from one site to another), allografts (same species tissue transferred to another), and xenografts (same species tissue transferred to other species) (Han et al., 2014a). It would be ideal to regenerate periodontal attachments, cementum, and alveolar bone as part of the optimal repair strategy for periodontal tissue defects. Researchers developed a tissue-engineered “sandwich” complex (a tissue-engineered membrane sandwiched between two mineralized tissues) to heal periodontal defects. Beagles’ premolar areas were treated using periodontal membranes. The sandwich tissue-engineered complex effectively healed periodontal abnormalities 10 days after surgery. Optimal periodontal repair may be achieved with tissue-engineered sandwich complexes, showing a mandibular defect treatment in a dog model (Wu et al., 2018). Octa CaP (OCaP) and collagen composites (OCaP-collagen) have boosted bone healing after cystectomy in humans. A canine or mouse model with a critical-size lesion benefited from OCaP-collagen-mediated bone regeneration (Kawai et al., 2017). Kawai et al. (2017) reported that OCaP-collagen was successfully used to restore a human mandibular bone defect, suggesting that it might be a viable bone replacement material in patients with significant bone defects that do not heal spontaneously. Schweikle et al. (2018) demonstrated that pH could efficiently regulate gelation time without affecting the flexibility of hydrogels. Hydrogels made of hybrid poly(ethylene glycol)-co-peptides are flexible platforms for bone regeneration. *In situ* gelation can be enhanced by hybrid PEG-peptide hydrogels. The increased structural characteristics of maleimide functionalized hydrogels and their enhanced tolerance to low pH makes them a preferred solution for injectable applications (Schweikle et al., 2018). Du and his colleagues (2015) coated nano-hydroxyapatite/coralline (nHA/coral) blocks with recombinant human VEGF by physical adsorption. Following the random implanting of VEGF/nHA/coral and nHA/coral blocks into a mandibular defect model, histomorphometric, immunohistochemical, and histological studies were performed to evaluate the healing process. nHA/coral blocks are suitable scaffolds for block grafting in critical-size mandibular defects, and angiogenesis can be enhanced by additional VEGF coating that can act through physical adsorption in the early stages of bone healing that present them as valuable biomaterials for bone healing (Du et al., 2015). It was the first time that gold nanoparticles were incorporated into a CPC (GNP-CPC), and the potential for osteogenesis was measured with human DPSCs. Furthermore, GNP-CPC significantly improved the osteogenic activities of hDPSCs. Through nanotopography and a bioactive addition, GNPs can enhance bone regeneration (Xia et al., 2018). Chitosan and glycerol phosphate composite (CS/-GP) was used by Cui et al. (2014) during bone regeneration with a thermosensitive transition in the sol-gel phase. Initially, ST2 stromal cells adhered to the CS/GP composite membrane better than CS alone. A significant difference between CS and GP composite membranes was observed in cell proliferation and osteoblast differentiation (*p* 0.05). CS/GP composite membrane implanted during surgery resulted in a mild inflammatory reaction, with no foreign body reaction lasting 4 weeks after surgery. Finally, the CS/-GP composite membrane was bioactive properties for bone tissue regeneration *in vivo* and *in vitro*.

#### 4.3.5 Chitosan therapy in craniofacial diseases

Chitosan (CH) is a deacetylated derivative of chitin, consisting of N-acetylglucosamine and glucosamine. The polysaccharidechitosan has free amino and hydroxyl groups, and can incorporated with other bioactive compounds. Because of its biocompatibility, biodegradability, low toxicity, and controlled dissolution by enzymes, chitosan is commonly used in tissue engineering as a biomaterial (Chang et al., 2017). According to Raisi et al. (2010), chitosan conduits improve peripheral nerve regeneration in rats suffering sciatic nerve transections. According to the researchers, the chitosan conduit might be used in clinical settings for peripheral nerve regeneration because it is easy to access, inexpensive, simple to produce, and biodegradable. Furthermore, it does not contain any foreign ingredients that may cause an allergic reaction in the human body. Therefore, this scaffold shows promising results for the sciatic nerve and craniofacial injuries, which might cause facial nerve damage. Using experimental injectable chitosan hydrogel, Moreira et al. (2021) expected that PBMT would not interfere with the distribution of endogenous stem cells in the dental pulp to result in a suitable reconstruction. Researchers used chitosan hydrogel in combination with a PBMT and blood clot and discovered that dental pulp regeneration results could be improved. Tsao et al. (2011) developed a new polyelectrolyte hydrogel composed of chitosan-PGA, which can be used as a wound dressing. Chang et al. (2014) used this scaffold to stimulate the healing of the alveolar socket after tooth extraction. Each rat’s extraction socket was examined at 1, 2, 4, and 6 weeks following extraction. Radiography and histology indicate that C-PGA-treated extraction sockets grew lamellar bone (6.5%) 2 weeks following extraction. Furthermore, they conclude that C-PGA holds great promise in promoting new alveolar bone growth. Posnick and Sami (2015) examined the results of 50 patients treated with interposition grafting and Le Fort I in a 3-year timeline. It was necessary to use an interpositional graft for maxillary repositioning because horizontal advances and vertical and transverse changes had to be made. All the implants were made of corticocancellous (iliac) bone. The analysis of each patient’s maxillary vector change points was done using an analytical model. According to the current study, iliac corticocancellous allografts have few systemic problems and are used to replace complex 3D bone defects resulting from Le Fort I osteotomies or relocations. A person’s overall health needs to include identifying and treating extra-skeletal conditions. Medical treatment is usually available for endocrinopathies. Females suffering from precocious puberty may benefit from the usage of Letrozole, an inhibitor of the aromatase enzyme. An aromatase inhibitor and an antagonist of testosterone receptors are usually needed by men instead. Analogs of somatostatin, such as pegvisomant, an antagonist of growth hormone receptors, benefit patients with excess growth hormone (Feuillan et al., 2007; Boyce et al., 2012; Salenave et al., 2014). At present, no medicines have been proven to be effective for FD. Craniofacial fractures are still treated most effectively with surgery. Sixty-eight percent of procedures result in regrowth, especially when growth hormone excess is not treated, so an endocrinology evaluation and treatment can be recommended before surgery (Boyce et al., 2016). Bisphosphonates are often prescribed to patients with craniofacial FD who experience discomfort (Burke et al., 2017).

#### 4.3.6 Drug therapy in craniofacial diseases

Zebrafish have proven to be an excellent model organism for *in vivo* drug testing. Zebrafish are well-known for their genetic tractability, significant fertility, external embryonic development, large clutch size, and visual transparency as vertebrate models (although newly discovered compensatory mechanisms and gene redundancy suggest zebrafish are redundant as genetic models). The ability of zebrafish embryos to diffuse oxygen is a critical feature for studying the development of drugs *in vivo* since drug treatments that alter cardiovascular system formation are likely to influence embryo survival (Wiley et al., 2017; Seda et al., 2019). Orofacial clefts (OFC) have been linked to valproic acid (VPA), an anti-epileptic medicine. Gebuijs et al. (2020) investigated how VPA affects bone and cartilage development in zebrafish larval heads in the early development stage (1–13 h after fertilization) and the late development stage (25–37 h after fertilization) when cranial neural crest cells (CNCCs) start arising, increasing, and differentiating. Despite the bone and cartilage absence, the body compensates by forming other tissues. A central mechanism of VPA causing craniofacial deformities (CDFs) seems to be the disruption of CNCC activity, leading to abnormal cartilage and bone growth. Furthermore, the army has learned to treat acute hearing loss as early as possible on the battlefield to avoid further complications. Anti-oxidants and steroids have proven to reduce the risk of additional damage after an explosion by combating free radicals created during noise-induced hearing loss (Rajguru, 2013). Thrombosis of the vascular system prevents medication concentrations from being adequate at the site of infection, which results in ineffective medical therapy. A systemic abnormality needs to be corrected, such as diabetes management, immunosuppressant withdrawal or modification, or corticosteroid administration. Surgical intervention (sequestrectomy and debridement) and adjuvant therapy are used in conjunction with antifungal treatment. The best antifungal agent is AmB treatment (Ferguson, 2000). P. Spicer et al. (2013) used gelatin hydrogel substrates combined with poly(dl-lactic-co-glycolic acid) (PLGA) particle carriers to develop antibiotic-releasing, porous polymethylmethacrylate space maintainers. Three formulations with two different releasing speeds were tested: 1) burst and 2) prolonged. They were evaluated in different dosages *in vivo* on rabbits whose mandibular bone was infected with *Acinetobacter baumannii* (2 × 10^7^ CFU/ml-1). The results revealed that implants with a high-dose prolonged release had better effects on soft tissue healing than implants with a fast release. Implants with an extended release of colistin through PLGA microparticle carrier demonstrated enhanced soft tissue treatment compared to implants with the fast release of colistin in a gelatin-based carrier (Spicer et al., 2013). Many treatments are available to treat craniofacial diseases, including drugs, surgery, stem cells, and bioactive materials. The development of fundamental and predictable periodontal regeneration techniques has been documented since the 1980s. For periodontal disease treatment, graft materials are used to replace the bone loss caused by the disease, barrier membranes are used to prevent tissue regeneration, and bioactive chemicals stimulate tissue regeneration (Han et al., 2014a). In tissue loss, trauma, periodontitis, developmental, dental caries, pulpitis, or oral cancer illnesses, allograft or autograft bone is used to fill the hole created by lesion excision. Clinical researchers and clinicians are interested in regenerative dental tissue or tissue engineering for potential functional restoration following tissue volume repair (Xu et al., 2021). Silver diamine fluoride (SDF) is an effective substance for non-invasive therapy. SDF is one of five breakthrough treatments recognized by the Food and Medicine Administration (FDA), which could be the first FDA-approved caries treatment (Horst, 2018). The current biomaterial and synthetic materials therapy in craniofacial and dental trauma and disease are summarized in Table 3.

**TABLE 3:** Current biomaterial and synthetic material therapy in craniofacial diseases.

Opinion	Materials and methods	Results	Ref.
Leukocyte and PRF	Evaluating the possibility of postoperative and intraoperative effects of L-PRF in PAOO	PAOO makes use of L-PRF quickly and safely. Combined with traditional bone grafts over 2 years, it may speed up wound healing and reduce the infection, pain, and inflammation after surgery	Munoz et al. (2016)
Bovine-derived xenograft and bioactive glass	A bovine xenograft and bioactive glass were compared for treating mild crowding in adult patients with PAOO	The combination of orthodontic therapy and periodontal surgery may be an effective treatment for adult patients to minimize the time and risk of root resorption. Bovine-derived xenografts with modified corticotomies enhanced bone density better than bioactive glass	Bahammam (2016)
Collagenated cortico-cancellous porcine bone	Micro-CT and histology assess the healing of porcine bone grafted into human extraction sockets over time using synchrotron radiation X-ray microtomography	Porcine bone is suitable for maintaining post-extraction sockets based on the morphometric findings	Giuliani et al. (2018)
PRP	A study was conducted to see whether PRP injections would improve the appearance of photodamaged skin and its texture and color	According to masked volunteers, a single treatment with PRP improved fine and coarse texture more than regular saline. Both participants and raters determined that PRP was marginally but not significantly better than normal saline	Alam et al. (2018)
HA particle/poly-L-lactide (HA-PLLA) composite	Using computational fluid dynamics, researchers simulated peak von Mises stresses (PVMS) and deformation of bone plates made of four materials: PLLA, PLLA, titanium alloy, and magnesium alloy. A fracture surface runs from the left mandibular angle on the 3D virtual mandibular model	Clinical settings prefer HA-PLLA over PLLA because of its reduced deformation. Improving the quality of HA-PLLA fixation can be a promising alternative to be used in craniofacial surgery and face bone trauma	Park et al. (2020b)
NGF	A study of endogenous NGF and its receptor tropomyosin receptor kinase A (TrkA) found that they peak during the cartilaginous reconstruction of a tibial fracture. Using two injection regimens, the researchers found that regional-NGF injections during the endochondral/cartilaginous phase increased bone formation markers	These findings report that NGF boosts cartilage-to-bone transition in a mouse model and identifies pathways that may assist researchers	Rivera et al. (2020)
PVA-PCL-HAB	This study developed PVA-PCL-HAB, a nanocomposite scaffold made of PCL-PVA-hydroxyapatite-based bioceramics. In combination with a hydrophobic PCL, a hydrophilic PVA scaffold generated by dual electrospinning of PCL and PVA may enhance the osteoconductive properties of PCL	Specifically, HAB-PCL-PVA scaffolds facilitate the attachment and development of stromal stem cells (MSCs from human bone marrow and DPSCs). *In vitro*, the scaffold promoted osteogenic differentiation, and *in vivo*, it promoted vascularized bone growth. This could lead to using the PVA-PCL-HAB scaffold in dentistry and orthopedics	Prabha et al. (2018)
PS-SAG-VEGF-BMP	A study was conducted to determine if cross-linked polysaccharide-based scaffolds containing smoothened agonist BMP-6 and VEGF might significantly enhance bone regeneration	Using the FDA-approved recombinant human BMP2 as a growth factor, agonist stimulation of the hedgehog signaling pathway improved craniofacial bone healing *in vivo*. Furthermore, drugs targeting this route could offer a novel reconstructive option for bony CFDs to treat non-union and delayed-healing fractures	Miller et al. (2019)
Teriparatide	According to the researchers, intermittent administration of recombinant parathyroid hormone (teriparatide) reduces fibrosis and increases osteogenesis in the graft environment	This method revealed that teriparatide-treated animals produced substantially more bone matrix than control animals. Teriparatide appears to reduce scarring, leading to better bone transplant integration	Cohn Yakubovich et al. (2017)
Volume-stable collagen scaffold (VCMX)	Immunohistochemistry was used to assess the effect of a VCMX on periodontal regeneration	Both pristine and regenerated PDLs showed a high cell turnover following VCMX treatment. Using this VCMX, periodontal healing was enhanced in intrabony deficiencies	Imber et al. (2021)
Chitin-PLGA/nano bioactive glass-ceramic (nBGC)/cementum	As a result, the cementum, PDL, and alveolar bone layers consist of chitin-PLAGA polymers (PLGA) and nano bioactive glass-ceramics (nBGCs)	Finally, nanocomposite hydrogel scaffolds containing growth factors may be used for complete periodontal regeneration instead of conventional bone grafts	Sowmya et al. (2017)
Dispersing iPSMSC-microbeads in CPCs	Using iPSMSC-microbeads dispersed in CPC, we developed a novel tissue-engineered construct that could be injected into a bone model to investigate bone regeneration; we developed an injectable cell delivery system using encapsulated iPSCMSCs. To study bone healing *in vivo*, disperse iPSMSC-microbeads in CPC	A novel injectable CPC-microbead-cell construction, BMP2-iPSMSCs, and OS-iPSMSCs stimulated bone regeneration as much as 2–3 times that of CPC controls. iPSMSC-CPC-BMP2-iPSC provided seven times more scaffold resorption than CPC control. Therefore, CPC-microbead-iPSMSC may be used in orthopedics, dentistry, and craniofacial surgery to promote bone regeneration	Wang et al. (2016a)
CPC-PLGA	In terms of *in vivo* performance, the researchers evaluated the performance of CPC-PLGA with or without CMC *versus* devitalized bovine bone mineral (DBBM) predicate Bio-Oss^®^	The study found that after a 26-week implantation period, CPC-PLGA generated good bone responses with >90% breakdown and >40% new bone formation	Grosfeld et al. (2016)
Hybrid poly(ethylene glycol)-co-peptide hydrogels	pH can control gelation time without altering the flexibility of the hydrogels produced. A lower pH level results in denser cross-linking in maleimide-functionalized PEG gels than in vinylsulfone-functionalized PEG gels	According to the results, PEG/peptide hybrid hydrogels are helpful for *in situ* situations. Like low pH tolerance, their unique characteristics can be an attractive solution for injectable applications	Schweikle et al. (2018)
Recombinant human VEGF	The nano-hydroxyapatite/coralline (nHA/coral) block grafting was used to optimize the regeneration effect of the canine mandibular critical-size defect model. At that point, rhVEGF165 was physically coated onto the nHA/coral blocks to increase the regeneration efficiency	nHA/coral vascularized blocks may provide a viable possibility for block grafting in large-scale alveolar defects. Additionally, in the early stages of bone healing, physical adsorption of VEGF promotes angiogenesis, providing significant potential for revascularized nHA/coral blocks as a bioactive material	Du et al. (2015)
PEGylated stearyl amine (pegSA) lipoplexes	Prevascularized nHA/coral blocks may prove ideal scaffolds for block grafting in critical-size defects in the mandible. Physical adsorption of additional VEGF may promote angiogenesis during the early stages of bone healing, implying significant growth potential for bioactive materials for bone regeneration in large-scale defects in the mandible	By expressing BMP-9 in C2C12 cells, lipoplexes were shown to promote osteogenic differentiation, as evidenced by increased calcium deposition *in vitro*, demonstrating the possibility of lipoplexes for bone regeneration. Furthermore, *in vivo*, acute toxicity testing has shown that the lipoplexes produced are safe. *In vivo* preclinical testing of PegSA lipoplexes is promising for future proof-of-concept studies	Vhora et al. (2018)
gold nanoparticles (GNP-CPC)	CPC containing gold nanoparticles is known as GNP-CPC. DPSCs from humans were studied to see if they could induce osteogenesis	Additionally, GNP-CPC significantly enhanced osteogenic activity in hDPSCs. Adding nanotopography to CPC and acting as bioactive additions to GNPs can improve bone regeneration	Xia et al. (2018)
BGC	3D-printed BGC scaffolds containing elements (Cu, Fe, Mn, Co) with photothermal effects and osteogenic differentiation potential were created. Furthermore, these scaffolds were also thoroughly examined for their photothermal anti-tumor and osteogenic activity	Using dual-functional bioactive ions with tissue engineering scaffolds could be an effective strategy for treating bone tumors, as these scaffolds show promise for the photothermal treatment of bone tumors and bone regeneration	Liu et al. (2018)
Cryogel	To demonstrate the feasibility of the production of site-specific implants to examine bone regeneration, patient-specific 3D prints were obtained together with cryogel scaffold manufacturing	Regardless of the mold shape, all cryogels were crushable without fracture propagation. The 3D nature of these problems and the scarcity of donor bone in children makes a patient-specific scaffold ideal for pediatric clefts	Hixon et al. (2017)
Bone graft	This study aimed to fabricate tissue-engineered bone grafts and test them on an alveolar cleft model to determine their ability to stimulate osteogenesis	An evaluation of tissue-engineered bone transplants before their use in a clinical setting was appropriate for this research design. The experimental setup with the stated maxillary defect did not show a boosting effect of bone grafts containing bHA	Korn et al. (2017)
DPSC	DPSC development and differentiation were tested with PuraMatrix^TM^	In PuraMatrix^TM^, DPSC continued to survive and proliferate for at least 3 weeks. Microscopy evidenced that, in some instances, cells grown in PuraMatrix^TM^ had healthy cell morphology, including cytoplasmic elongation. The DPSC cells produced DMP-1 and DSPP in tooth slices containing PuraMatrix^TM^ after 21 days	Cavalcanti et al. (2013)
Autograft scaffold	Human dental pulp was decellularized in three ways to make it a viable scaffold for autografts	According to this study, it is possible to decellularize human dental pulp and produce a scaffold that promotes SCAP proliferation and differentiation	Song et al. (2017)
Autologous grafts	An engineered cartilage transplant was performed to see if it could safely and effectively restore the alar lobe	It is possible to generate and use autologous nasal cartilage tissues therapeutically for the functional repair of alar lobules. These engineered cartilages are considered a promising alternative for facial defects	Fulco et al. (2014)
Polycaprolactone-tricalcium phosphate (PCL-TCP)	MSCs seeded into PCL-TCP successfully repaired a vertical X-shaped bone defect in a dog’s jaw. Using differentiation labeling procedures and flow cytometric analysis, aspirated bone marrow from a dog humerus was examined	The right and left sides were treated differently as a control side defect, the left with pure PCL-TCP scaffolds and the right with cell-loaded scaffolds. After 8 weeks, a histomorphometric examination showed more lamellar bone on the tested side than on the control side (left side). The study results revealed that PCL-TCP could effectively affect MSC loading in bone repair	Khojasteh et al. (2013)

### 4.4 Soft tissue regenerative medicine in craniofacial diseases

#### 4.4.1 Regenerative medicine in facial organ injuries

The pinna hematoma should be incised and drained immediately to prevent cartilaginous hematoma. It is common for the tympanic membrane to rupture, and it typically happens in conjunction with other injuries, more severe. Blast injury should be considered in every service member exposed to blast injuries, even if there are no prominent head, face, or neck injuries. An audiogram should be performed after other injuries have been ruled out. The war conditions necessitate aid, even when most perforations heal independently. One study found that five of 14 membranes left untreated did not heal after the bombing of the US embassy in Kenya, whereas all those treated did (Rajguru, 2013). Research has shown that porous polyethylene repair will provide better aesthetic results, shorter surgical times, fewer treatments required, and a faster postoperative recovery than autologous reconstruction (Ali et al., 2017). Medpor’s ability to contour and mold the polyethylene implant intraoperatively allows for a good fit with the contralateral ear in most instances following auricular reconstruction (Wilkes et al., 2014). Fractures and hematomas in the septum can result from nasal trauma. Septal hematomas require immediate incision and drainage to prevent infection and cartilaginous necrosis. The treatment plan for epistaxis should include anterior and, if needed, posterior nasal packing. The anterior packing of the nose can be done with Merocel or Net cell nasal packs and ribbon gauze soaked in antibiotic solution. Foley catheters of 12G or 14G should be inflated to three-quarter capacity in the nasopharynx, then dragged anteriorly until they impact posteriorly. It is possible to remove the packs and reevaluate the situation in 24–48 h (Rajguru, 2013). Gentile et al. (2016) used autologous chondrocyte micrografts and platelet-rich plasma (PRP). The utilization of chondrocyte micrografts produced from the nasal septum poured PRP in solid form was proven to regenerate the cartilage of external nasal valve collapse in this study.

Reconstructing the lip requires realigning anatomical markers and restoring functional muscle and sensory function. Unless the affected portion of the lip covers more than 30%, a primary closure may be attempted. Skin grafting may be an option and local advancement flaps, but it is best to repair the defect with accessible lip tissue (Braun and Maricevich, 2017). According to Grewal et al. (2021), two-stage cleft lip surgery improves facial symmetry and final lip, nose, and palate restoration. This research supports their theory that a two-stage repair can provide functional and aesthetically pleasing results instead of presurgical nasoalveolar shaping. Vitagliano et al. (2016) described a new lip-repair procedure involving nasolabial flaps and adipose tissue transplants in a single study. It has been concluded that surgical removal of a tumor alone does not restore facial eurythmic. Transplanting adipose tissue can accomplish this. Lip repair can be significantly improved functionally and cosmetically with these two methods, ensuring excellent long-term results. According to Pushpoth et al. (2008), AlloDerm is a helpful lamellar graft used in treating full-thickness lid deformities. The potential for AlloDerm to be an excellent posterior lamellar replacement is relatively high when there is enough skin cover to drape over it. Conjunctiva grows smoothly over its structure because it replaces the tarsus and acts as a scaffold.

#### 4.4.2 Regenerative medicine in skin injuries

The first skin replacements were made from porous matrices mimicking the dermis and serving as dermal regeneration templates. In addition to enhancing wound healing, these matrices can be used to deliver medications and aid in regenerative cell adhesion. Dermal scaffolds or autografts of epidermal keratinocytes were previously implanted (Borrelli et al., 2020). Certain GF families may enhance wound healing if correctly used because they are involved in endogenous wound healing. In a double-blind clinical study, epidermal growth factor (EGF) was applied topically to skin transplant recipient sites and significantly enhanced epidermal regeneration (Brown et al., 1989). For wound dressings, Oh et al. (2016) employed an electrospinning process to create composite fibrous mats made up of chitosan (CH), poly(caprolactone) (PCL), or chitosan-caffeic acid conjugate (CCA). These findings suggest that PCL/CCA fiber mats can be used in skin tissue engineering. The nanoparticle-in-nanofiber system developed by Xie et al. (2016) for wound healing applications releases two growth factors simultaneously. The nanofibrous meshes mimic the natural healing process, but they support it by electrospinning chitosan and poly(ethylene oxide). Angiogenesis was enhanced by nanofibers containing vascular endothelial growth factor (VEGF).

Additionally, poly(lactic-co-glycolic acid) (PLAGA) nanoparticles encapsulated with PDGF were injected into nanofibers to promote organ regeneration and tissue remodeling. Our findings indicate that nanoparticles embedded in nanofibers might be a therapeutic tool for chronic and routine wound healing (Xie et al., 2013). Similarly, Guo et al. (2015) found that the attractive chemokine SDF-1/CXCR4 is involved in epidermal stem cell migration during wound healing. A significant increase in SDF-1 levels was seen at wound edges after damage, and CXCR4 was examined in epidermal stem cells and proliferative epithelial cells. An experiment conducted by Kim et al. (2011) examined whether adipose-derived stem cells (ADSCs) have the potential to reverse the signs of aging, in particular by increasing dermal collagen production and angiogenesis. By growth factor production and fibroblast activation, it has been shown that ADSCs have wound-healing, whitening, and antioxidant properties on the skin. According to their findings, using ADSCs for treating age-related skin deterioration was useful. Clinically, the aim is to develop injectable nanocomposite conductive hydrogel dressings that can function as adhesive dressings, antibacterial, and radical scavengers and have superior mechanical properties that improve the healing of full-thickness skin wounds. Liang et al. (2019) made wound dressings from HA-grafted-dopamine and reduced graphene oxide (Dominiak et al., 2020) using an H2 O2/HPR system. Therefore, they are ideal wound dressings for promoting full-thickness skin restoration thanks to antioxidative, conductive, and adhesive hydrogels with sustained drug-release properties.

#### 4.4.3 Regenerative medicine in nerve and muscle injuries

Surgical removal of a parotid tumor, trauma, or petrous bone surgery can injure the facial nerve or be absent at birth. An abnormality of the facial nerve can cause functional movement difficulties and facial asymmetry, significantly lowering the quality of life (Borrelli et al., 2020). A minimally invasive liposuction process can be used to obtain ADSCs that are useful in nerve regeneration Watanabe et al. (2017). According to the results, both undifferentiated and differentiated ADSCs have therapeutic potential for facial nerve regeneration when used in a cell-based treatment. A mouse model with severe axon damage showed dramatic functional recovery from sciatic nerve crush after ADSCs was administered intravenously 1 week after the injury. Marconi et al. (2012) reported that the regeneration of nerves might also be assisted by other cells besides ADSCs. bFGF stimulates the growth of proximal axons from the stump of the proximal nerve and impacts nerve cells, Schwann cells, and fibroblasts, which play an essential role in the regeneration of the peripheral nerve. Matsumine et al. (2016) developed acidic gelatin hydrogels to deliver bFGF. They aimed to determine whether it would release bFGF and trigger peripheral nerve regeneration *in vivo* for many weeks. Seven weeks after implantation, histology revealed a significantly higher rate of regeneration, the induction of a large number of newly developed nerve axons, and a higher rate of mature nerve axons in the BGF microsphere group than in the BGF-free group.

### 4.5 Hard tissue regenerative medicine in craniofacial diseases

#### 4.5.1 Regenerative medicine in the frontal process of the maxilla (FFPM)

Traditional treatment ways for FFPM include open reduction and internal fixation with bone plates or wire. Although these procedures are faster than surgery, some drawbacks exist, such as more significant trauma, scars on the face, and a more extended recuperation period. Meng et al. (2020) performed fracture reduction using an endoscopic endonasal procedure, a novel surgical technique. It provided good exposure to the FFPM. FFPM reduction provides an anatomical reduction with an outstanding aesthetic result, a straightforward process, and a visible operation. FFPM can be treated more effectively with this less invasive method.

#### 4.5.2 Regenerative medicine in maxilla and mandible

Open reduction of craniofacial bone fractures generally requires metal plates and screws. Alternatively, metal implants may result in protrusion, palpation, or a foreign body response, requiring another procedure after removal. Metal implants have recently demonstrated promising results when combined with absorbable implants. The absorbable mesh and screws employed by Ju et al. (2019) were utilized to decrease the open fractures and fragments of bone. Maxillary fractures can be treated with absorbable implants and screws without requiring additional surgery to remove the metal plate. Furthermore, fixing small bone pieces with cyanoacrylate is an exact and secure process, meaning that screws cannot be used. Chen et al. (2021) studied a midline palatal suture’s fracture mechanics and stress distribution during surgically assisted fast palatal expansion in maxillary transverse deficit under dynamic stresses. CBCT data from a 21-year-old female volunteer were used to create a model of the CMF complex (including the palatal suture). A finite element analysis model was developed based on meshwork. In order to determine the time-load curve, palatal suture yield strength was obtained within 140 m after a force was applied that increased from 0 to 500 N, simulating non-surgical bone expansion (model A). This approach examined the fracture process, time, and stress distribution of the palatal suture in both maxillary lateral osteotomies (model B) and LeFort osteotomy I-assisted (model C) extension of the maxillary arch. In the model B arch expansion with lateral osteotomy, the palatal suture rupture and lateral maxillary extension were comparable to the arch expansion with LFIO (model C). Maxillary lateral wall-osteotomy may be an alternative to LFIO due to its stress and significant consequences. Physiological and structural changes following extraction threaten the integrity of the anterior maxilla’s bone walls. A regenerating strategy is essential to enable hard and soft tissue dimensions to be preserved throughout time. Chappuis et al. (2018) observed 20 patients over 10 years to examine the impact of early implant placement with simultaneous contour augmentation by guided bone regeneration. This study revealed an effective technique for early implant placement and contour augmentation using a two-layer composite graft in post-extraction single tooth sites, resulting in stable bone conditions and low mucosal recession risks over a 10-year observation period. Orthodontic treatment is not automatically recommended when the maxilla and the mandible have severe sagittal differences. Such cases require the participation of a surgeon, an orthodontist, and a periodontist, coordinating an interdisciplinary approach. Each patient’s treatment is tailored to their specific needs and developed individually, regardless of the use of conventional treatment approaches. A variety of complications may arise from the surgical correction of bone abnormalities of the jawbones. Orthognathic abnormalities can now be treated using RM, already commonly used in reconstructive surgery. Using allogenic 3D graft blocks as an alternative for issues during regular orthodontic treatment, Dominiak et al. (2020) uncovered a technique for reconstructing sagittal alveolar bone with a 3D reconstruction style. High-frequency acceleration (HFA) promotes alveolar bone growth in physiological settings and during the healing process following tooth extractions. Alikhani et al. (2019) investigated HFA’s impact on the osteoporotic alveolar bone, finding that it acted as a regenerative agent. Vibration enhanced anabolic metabolism and reduced catabolic metabolism in the alveolar bone of osteoporotic rats. Their study suggests that HFA could be a potential therapy in patients with low alveolar bone density due to osteoporosis. An investigation was conducted by Pourebrahim et al. (2013) to assess bone regeneration following autogenous bone transplantation and stem cells obtained from adipose tissue in canine maxillary alveolar clefts. From under the skin of a dog, MSCs were extracted. In addition to autograft, tissue-generated bone seems a viable alternative to bone regeneration. Common congenital abnormalities include cleft lip and palate (CLP). It can occur unilaterally or bilaterally and has acquired and hereditary causes. Alveolar cleft grafting remains contentious regarding time, materials, and surgical procedures. Alveolar cleft defects can be repaired by several grafting strategies, according to Mossaad et al. (2019). Artificial iliac crest bone grafting has proven more reliable than using nano calcium hydroxyapatite or BMSCs as substitutes. Using human dental pulp stem cells (DPSCs) obtained from deciduous teeth, Jahanbin et al. (2016) investigated the ability of DPSCs to regenerate maxillary alveolar lesions in Wistar rats. In rats, human DPSCs provided a viable model for restoring maxillary alveolar defects in CLP patients, providing an extra resource of cells for healing. In another investigation, Feng et al. (2016) used MSCs to treat the bone regeneration of mandibular distraction osteogenesis in rabbits plus Runt-related transcription factor 2 (Runx2). They found that rabbit mandibular distraction osteogenesis was efficiently enhanced, and the fixed period was reduced using Runx2 *ex vivo* gene therapy. Therefore, there would benefit from repairing craniofacial fractures. The osteogenic drug dipyridamole incorporated into 3D-printed bioceramic scaffolds is effective for critical-sized calvarial lesions healing in skeletally mature translational animals. Yet, no studies have developed craniofacial models with this design. Maliha et al. (2020) implanted bioceramic/dipyridamole scaffolds into a developing calvaria limb and measured bone formation according to geometry and dipyridamole concentration. The suture’s growth may be adversely affected and aimed to improve bone formation in children using a pediatric scaffold and a dipyridamole concentration to maintain the cranial suture. The condition of osteoporosis results in an increased risk of fractures as the structural integrity of bone tissue deteriorates. Bone tissue engineering procedures typically utilize calcium phosphates (CaPs), which are similar to bone apatite, except that they do not contain any trace elements (TE). Bioactive glass is used in dentistry and CMF surgery due to its capacity for bonding with bone and stimulating osteoblastic development. Using rat bone marrow mesenchymal stem cells (BMMSCs) from osteoporotic and normal animals, Chen et al. (2017) studied the osteogenic effects of quaternary Si-Sr-Zn-Mg-cooped CaP or 45S5 BG ionic dissolution products. The results indicate that TE-CaP and 45S5 BG work together to promote osteoporotic and normal growth and differentiation of rMSCs.

#### 4.5.3 Regenerative medicine in orbital floor

Orbital fractures possess a unique trauma mechanism because of the complex architecture of the bones and soft tissue elements involved. An orbital blowout fracture can increase the orbital volume, causing conjunctival hypertrophy and enophthalmos. Other cases may require surgical intervention, even if conservative methods are used (Koenen and Waseem, 2021).

#### 4.5.4 Regenerative medicine in periodontal diseases

Several treatments and medicines can be used to eliminate or alleviate periodontal disease. Plague-induced gingivitis is prevented by mechanical cleaning, removing plaque, and cleaning teeth. Periodontal disease and its damaging effects cannot be treated with this approach. Alternative treatments are needed to achieve better results. Mouthwashes and dentifrice, for instance, act as plaque-removal agents. In addition to mechanical methods, these products can also be used. Statistically significant reductions in plaque and gingivitis were shown in one study (Teughels et al., 2013). According to a second study, the best dentifrices reduce plaque most effectively when they contain chlorhexidine and triclosan (Faggion et al., 2014). The tooth brushing technique can be used in addition to other mechanical practices, such as subgingival debridement. Nevertheless, it has several limitations; for example, it cannot be used in all situations. When people who smoke regularly recover from surgery or non-surgical treatment, their pocket depth decreases by 0.23–1 mm (Heasman et al., 2006). PDL fibers anchor the tooth in the socket by binding the cementum on the tooth root surface to the alveolar bone and reducing occlusal pressures. Regeneration of PDL is crucial to successful periodontitis treatment. A great result would be for the newly restored bone and cementum to be perpendicularly connected to the newly implanted collagen fibers (Liu et al., 2019). Bone loss resulting from periodontal disease can be replaced by guided bone regeneration (GBR). Several drawbacks to the GBR procedure include the requirement for a stable barrier membrane during surgery and a new creative cavity. Tsai et al. (2016) used an innovative microinjector to overcome these drawbacks and this innovation could provide bone graft replacements for the healing of periodontal disease bone deficiency through the use of a microinjector.

Dental caries is considered one of the most frequent chronic diseases in the United States, affecting 92% of people aged 20–64 years. An alternative technique for replacing damaged tooth tissues is scaffold-based tissue engineering. NF-gelatin/MAGP hybrid scaffolds were created based on nanofiber gelatin and magnesium phosphate using biomimetic techniques by Qu et al. (2014). DPSC proliferation, differentiation, and biomineralization are facilitated by these scaffolds that mimic the nanostructure and chemical composition of the natural dentin matrix. Developing biomimetic nanostructured hybrid scaffolds that release metallic ions regulated in a controlled manner may enhance the scaffolds’ ability to regenerate tooth tissues. Researchers showed that a new lipoxin analog (benzo-lipoxin A4, bLXA4) could induce the hard and soft tissue regeneration permanently lost to periodontitis. They concluded that NPRM-bLXA4 mimics endogenous resolving mechanisms and can be helpful in therapeutic tissue engineering for chronic osteolytic inflammatory diseases (Van Dyke et al., 2015). Multifunctional growth factors, including transforming growth factors-βσ ((TGFs), are important for many cell types. Hassan and Alagl (2015) used a bovine bone mineral matrix with TGF-1 for intrabony defect treatment compared to using only inorganic bovine bone, and they found it effective. According to their results, the sole usage of inorganic bovine bone was not helpful. A modern injection method using microporous CaP cement (CPC) and either fibroblast growth factor-2 (FGF-2) or BMP-2 was used by Oortgiesen et al. (2014) to determine the regeneration capabilities of the implant. Comparing CaP + FGF-2 with CaP + BMP-2, the combination therapy differed considerably in PDL repair score. Compared to CaP alone, FGF-2 + CaP led to significant improvements in bone healing, whereas CaP + BMP-2 did not demonstrate any significant changes. A possible periodontal regeneration therapy could combine a topical FGF-2 application with an injectable CaP. Tang et al. (2015) studied how bone resorption in experimental periodontitis rats was affected by osteoprotegerin (OPG) gene therapy. We examined the effects of OPG on alveolar bone protection using an experimental periodontitis model in which a bacterial plaque-retentive silk ligature was placed around the maxillary second molar tooth, *Porphyromonas gingivalis* (a bacterial plaque-retentive bacteria) was injected, and a high-carbohydrate diet was consumed. Experimental periodontitis decreases alveolar bone height in rats, and local recombinant OPG gene therapy slows it down. The prevention of periodontal bone loss might be possible through OPG gene therapy. This study found a novel regeneration therapy for teeth and periodontal defects and posttraumatic fractures of the alveolar bone in the place of the maxillary front teeth had been found by Kiyokawa et al. (2009). Dislocated teeth are removed first, followed by the removal of crushed alveolar bone. Reimplanting the displaced teeth is followed by grafting the surrounding bone (marrow) with a cancellous iliac bone to promote periodontal bone growth. This procedure offers a cosmetic and functional recovery superior to the standard treatment in situations like these because no good teeth nearby are sacrificed, and no-good teeth are lost. This can be applied in the future as a new treatment for alveolar fractures. Using mineral trioxide aggregates (MTA), apexification is now preferred for permanent anterior immature necrotic teeth. Apexification decreases the canal wall’s thickness and makes them short so that they are likely to cervical fracture because it prevents teeth from maturing and developing further. Yoshpe et al. (2021) introduced a new method called the regenerative endodontic procedure (REP) used in immature necrotic molars treatment patients. In addition to root extension and expansion, REP allows periapical healing in the dentinal root canals. They discovered that platelet-rich fibrin (PRF) with REP has more advantages than MTA apexification due to extending roots, thickening woody root canal walls, and narrowing the apical foramen. An osteoinductive protein/peptide may be employed with a nanofibrous extraction or bone graft to improve alveolar bone regeneration. Researchers at Boda et al. (2019) demonstrated that calcium-binding peptides mimicking BMP-2 could help regenerate periodontal bony structures. Mineralized nanofiber pieces, such as MF bone defect fillers, can offer a viable alternative to replace conventional dental bone graft formulations. Biological applications, such as soft tissue engineering, wound healing, and hemostasis, may benefit from these nanofiber pieces. Du et al. (2014) used allogeneic BMMSCs to create a tissue regeneration-inducted in a rat model. They suggested that injections of BMMSCs may work as anti-inflammatories and immunomodulators to reduce periodontitis-related abnormalities. Recent studies on RM in craniofacial and dental trauma and disease are summarized in Table 4.

**TABLE 4:** Regenerative medicine in craniofacial diseases.

Opinion	Materials and methods	Results	Ref.
Periodontal membrane made from tissue engineering	Using gingival fibroblasts as seed cells, the researchers looked at the ability of periodontal defects to heal and regenerate	By using the tissue-engineered sandwich complex, you may be able to complete your periodontal repair quickly	Wu et al. (2018)
Oleaginous calcium hydroxide suspension Osteora^®^ and Osteoconductive bone graft OssiFi™ (Equinox Medical Technologies, Holland)	This study examined the effect of osteoconductive bone graft in conjunction with OssiFi^TM^ osteostimulating oily calcium hydroxide solution Osteora^®^ (Metacura, Germany) on periodontal bone replacement in intrabony deficiencies	Periodontal intrabony defects may be treated with Osteora^®^ as an osteostimulating agent combined with osteoconductive bone grafts	Mahajan and Kedige (2015)
ADSCs and glue scaffold	A fibrin glue scaffold, both alone and combined with autologous bone grafts, was compared with fibrin glue scaffolds in rabbit mandibular lesions to test healing and regeneration	The thickness of cortical bone increased significantly as the healing process progressed when fibrin glue scaffolds plus ADSCs were utilized	Mehrabani et al. (2018)
Bone graft	An abnormal mandibular shape was corrected in a patient with plexiform ameloblastoma using autologous human bone marrow MSCs	An autologous human bone marrow MSC and autogenous bone graft were used to reconstruct the mandible of a patient with plexiform ameloblastoma, followed by an osseointegrated dental implant and prosthetics	Kim et al. (2013)
MSCs			
HBMSCs	Autogenous bone grafts and autologous human bone marrow mesenchymal stem were used for the mandibular defect. Following distraction osteogenesis, dental implants were placed, and prosthetic restorations were applied	The results revealed that human BMSC was successfully used for the mandibular defect	Shin et al. (2020)
Autogenous bone graft			
BMSCs + Bio-Oss	Bio-Oss grafting materials plus MBCaP were assessed without and with DPSCs and bone marrow-derived MSCs (BMSCs) in a rabbit calvarial defect model	In the study, autogenous bone was found to be the gold standard. Bio-Oss and MBCP both gained osteoconductive abilities when DPSCs and BMSCs were added. The results showed the same level of effectiveness as an autogenous bone after 8 weeks when BMSCs were mixed with MBCP and Bio-Oss. Based on these results, MSC-based bone tissue regeneration techniques and biomaterials are promising approaches	Shiu et al. (2021)
BMSCs + MBCaP			
DPSCs + Bio-Oss			
Fibrin-agarose biomaterials with cells	Acellular and cellular bone replacements were created using nanostructured fibrin-agarose biomaterials. MSCs derived from adipose tissue were differentiated to the osteogenic lineage. Afterward, the artificial tissues were tested in an immunodeficient animal model of a severe mandibular injury to see if they might aid in bone regeneration	The animal model of critically deficient mandibular bone shows promise, despite these preliminary results	Martin-Piedra et al. (2021)
AFAS FAOM	The scientists developed and created three new scaffolds called functionalized fibrin-agarose oral mucosa stroma substitutes (n-FAOM) and non-functionalized fibrin-agarose oral mucosa stroma substitutes (n-FAOM), and acellular fibrin-agarose scaffolds (AFAS). The components of the ECM were assessed by oral histochemistry and immunohistochemistry after 1, 2, and 3 weeks of *in vitro* development	*In vitro* tissue formation is improved by functionalizing the scaffold, improving its biomimetic aspects regarding ECM components, and reducing the amount of time it takes to prepare the scaffold	Blanco-Elices et al. (2020)
hDPSCs in cell sheets	When arranged in cell sheets CS, human DPSCs were tested to determine whether they could preserve their undifferentiated state and osteogenic differentiation capacity	Those with conditions that inhibit bone healing, like medication-related osteonecrosis of the jaw, diabetes, and osteoporosis, can benefit from the procedure	Pedroni et al. (2019)
ADSCs and SHED	A preliminary analysis of ADSCs and SHEDs secreted in their conditioned medium allowed both normal and osteo-inducing conditions to be maintained	Regulatory cytokines, chemokines, and growth factors profile of SHED were more pro-angiogenic than ADSCs	Mussano et al. (2018)
MSC in 3D-scaffold	In this study, BMMSCs and ADSCs were isolated and seeded on 3D-printed scaffolds and then transplanted into a rabbit model to heal mandibular osseous malformations	We report on the steps involved in creating a 3D-printed mandibular scaffold, the implantation procedure in rabbits, and microcomputed tomography (micro-CT) analysis after a significant mandibular bone defect is created	Fang et al. (2017)
ADSCs	An *in vivo* model of angiogenesis known as subcutaneous matrigel implants was used to examine the effect of ADSCs on nerve development	Based on these findings, ADSCs appear to contribute to nerve healing and development *via* BDNF production. By growing the cells in neural differentiation media before implantation, the stimulatory impact of the cells can be increased	Lopatina et al. (2011)
Devitalized pellets of engineered hypertrophic cartilage	A hollow cylinder of devitalized cancellous bone defined the size of a significant bone replacement. Devitalizing synthetic hypertrophic cartilage pellets made a bone-inducing substance. These pellets were either used alone or involved with SVF of adipose tissue as a source of osteoprogenitors and endothelial cells. Targeting vascularization of the area was achieved using an arterio-venous (AV) bundle inserted axially	Devitalized hypertrophic cartilage is osteoinductive to use off-the-shelf materials in the regenerative surgical technique	Epple et al. (2019)
MSC	A sheep’s AV-loop model was created with tricalcium phosphate-hydroxyapatite (TCP-HA) granules, and MSCs immediately auto-implanted	Future reconstructive procedures might be enhanced by this technique for creating vascularized, transplantable bone tissue	Boos et al. (2013)
and rhBMP-2			
SrFO	Lyophilizing chitosan, polyethylene glycol dimethacrylate, and β-tricalcium phosphate β-TCP manufactured by free radical polymerization, a semi-interpenetrating scaffold composed of SrFO was synthesized and loaded into biohybrid scaffolds	Therefore, the combination of stem cells from human dental pulp with SrFO is an up-and-coming method for regenerating osseous tissues under challenging conditions	Martin-Del-Campo et al. (2016)
TCP	One study on split-mouth goats used the two-wall bone to form two-wall alveolar clefts and repair them with autologous bone (the gold standard) on one side and β-TCP putty on the other	Thus, the findings of this study put β-TCP putty at the forefront of the therapeutic treatment of alveolar clefts and other complex two-wall bone defects	Janssen et al. (2017)
BMMNCs and PRF	The research was conducted to assess the efficacy of using autologous BMMCs in conjunction with PRF and nanohydroxyapatite for alveolar cleft repair	By combining BMMNCs, nanohydroxyapatite, and PRF, a new method can potentially replace traditional alveolar bone transplantation in alveolar cleft defects	Al-Ahmady et al. (2018)
Collagen plus stem cell	This study sought to determine how well autologous PDLSCs loaded into IMC could regenerate bone in minipigs with severe bone damage	Based on immunohistochemistry, both runt-related transcription factor 2 and Osterix were substantially expressed in IMC-produced neo-bones. The neo-bone generated by IMC was identical in nanostructure and nanomechanical properties to normal bone. IMC-based bone transplants are a potential therapy based on the findings of this research	Zhang et al. (2017)
GBR	Several strategies for guided bone regeneration (GBR) of peri-implant abnormalities were evaluated *in vivo* using an animal model	The peri-implant bone development will be improved with GBR treatments and CaP-coated implants. GFC and PDLSC did not significantly improve bone regeneration when used	Kämmerer et al. (2017)
hiPSCs	No studies comparing hiPSCs, hUCMSCs, and hBMSCs have yet been published that compare bone regeneration in these three cell types. These cells were considered suitable for bone tissue engineering and were tested for bone regeneration in rat cranial lesions after seeds were placed on biofunctionalized macroporous CPCs. Almost 90% of all three kinds of cells remained alive on CPC scaffolds	It seems that osteogenic genes are upregulated because mineral production by cells increases with time *in vitro.* Biofunctionalized macroporous CPC-stem cell constructs could regenerate bone more than twice as much as the cell-free control; hiPSC-MSCs and hUCMSCs represented viable alternatives to hBMSCs, and hiPSC-MSCs doubled the amount of new bone compared to the cell-free CPC control	Wang et al. (2015)
hUCMSC hBMSC	This study examined bone regeneration of hUCMSC and hBMSC seeded onto macroporous CPC in critical-sized cranial lesions in rats	hUCMSCs transplanted on CPC were shown to be as effective at bone regeneration as hBMSCs *in vivo*. Comparing hBMSC-CPC and hUCMSC-CPC constructions to CPC without cells, both produced more new blood vessels and bones. Using macroporous RhD-grafted CPCs with stem cell seeding has the potential as a craniofacial and orthopedic repair	Chen et al. (2013)
pBCP	Using heterotopic implantation in mice (mHI model) and rabbits (rabMCSD model), we investigated the bone-induction capacity of porous biphasic calcium phosphate (pBCP)	Researchers have discovered that pBCP could be an alternative to significant bone defect treatment and periodontal regeneration therapy in the rabMCSD model because it is osteoinductive and stimulates new bone and cementum-like tissue production	Santos et al. (2018)
TPP	Chitosan biopolymer, sodium tri-polyphosphate (TPP), and nano-hydroxyapatite (Boda et al., 2019) constructed porous spherical scaffolds. Cross-linking nHA/chitosan droplets with TPP was primarily achieved using ionic cross-linking	Live and dead cell assays were performed on both lyophilized and LSD scaffolds on the 14th day of *in vitro* experiments. Both scaffolds demonstrated excellent osteoblast adhesion and minimal cytotoxicity. Osteoblast attachment was higher on 2% nHA/chitosan scaffolds than on 0% nHA/chitosan scaffolds	Uswatta et al. (2016)
rhBMP9	Researchers aimed to determine whether HA worked in tandem with recombinant human bone morphogenetic protein 9 (rhBMP9), one of the most osteogenic BMP growth factors	These findings suggest that 1) HA may act as a carrier of diverse growth factors, and 2) rhBMP9 induces osteoblast development robustly and effectively. Additional animal studies are needed to examine this combined method *in vivo*	Fujioka-Kobayashi et al. (2016)
PLAGA	Exogenous VEGF supplementation and BMP-6 administration without MSCs were examined for their ability to repair bone. PLAGA microspheres were sinterable using thermal processes to produce 3D scaffolds (P)	VEGF and BMP-6 treatment for the bone defect enhanced angiogenesis and induced differentiation of endogenous MSCs by improving bone healing, unlike the delivery of either VEGF or BMP-6 alone	Das et al. (2016)
BMSCs	In the present study, lenti-GFP tracking was used to determine how porous silk scaffolds affected rat bone marrow stem cells (BMSCs) *in vivo* and *in vitro* in cranial bone lesions	It is shown that porous silk scaffolds provide favorable conditions for cells to grow, survive, and function for a long time to promote bone healing	Zhang et al. (2014)
rhBMP-2	This study assessed the effect of coating biodegradable, porous PPF scaffolds with CaP and simultaneously administering human bone morphogenetic protein-2 (rhBMP-2) on bone regeneration in living animals	Using rhBMP-2 administration in conjunction with a CaP-coated scaffold showed a synergistic impact that could potentially be used to regenerate massive bone defects	Dadsetan et al. (2015)
PCL	In this study, we created composite fibrous mats using electrospinning to fabricate CH, PCL, and chitosan-caffeic acid (CCA) for wound treatment	These findings suggest that PCL/CCA fiber mats can be used as biomaterials for wound dressings and skin tissue engineering	Oh et al. (2016)
SVF	An uncultured stromal-vascular-fraction (SVF) cell model of facial nerve regeneration was used here to study the ability of SVF cells to promote regeneration	Uncultured-SVF was infused into the neural conduit to result in optimum regeneration of the nerves, which was far superior to the nerve conduit alone	Matsumine et al. (2017)
Stem cell and autograft	Eighteen individuals with missing teeth and abnormal horizontal alveolar bone were tested in a randomized clinical trial involving individuals with traumatic damage and cleft palate. In addition to autogenous block grafts, patients had the option of stem cell treatment	Although stem cells can successfully treat massive alveolar defects, there are limitations to their ability to reconstruct significant alveolar defects fully. Additional refinement of this approach is needed to obtain results similar to existing approaches for treating significant abnormalities, especially cleft palates	Bajestan et al. (2017)
CPC scaffold containing cell-encapsulating hydrogel microfibers	Alginate-fibrin microfibers were constructed using partly oxidized alginate with varying fibrinogen concentrations (0%–0.8%) (Alg-Fb MF). On day 2, many cell-cell contacts were formed along the long axis of the microfibers in Alg-0.4% Fb MF. The fast-degradable Alg-0.4% Fb MF was mixed with CPC paste to create an injectable tissue-engineered construct for bone reconstruction	In an *in vivo* experiment, a CPC-MF construct encapsulated with hBMSCs showed strong bone regeneration properties. Compared to the control group, the CPC-MF-hBMSCs group had a fourfold increase in new bone area fraction in the rat mandibular defect 12 weeks after implantation	Du et al. (2015)
Three-component implant comprising autologous MSCs	The human demineralized bone matrix was transduced with an adenovirus vector that produces BMP-2	They accelerated osteogenesis by implanting BMP-2-modified MSCs in a dog model for bone distraction. This information will be used to guide future clinical trials involving mandible distraction	Castro-Govea et al. (2012)
Autologous cells enriched with CD90^+^ stem cells and CD14^+^ monocytes	The study involved thirty patients who needed augmentation of their maxillary sinuses. Stem cells were placed onto a β-triCaP scaffold or the scaffold alone for patients with 50%–80% maxillary sinus bone deficiency. Four months after engineering bone, clinical, radiological, and histological evaluations were performed. After the alveolar bone core is harvested, a dental tooth prosthesis is attached to the implanted alveolar bone	A 1-year follow-up showed no treatment-related adverse events in any of the patients who had oral implants and functionally loaded dental restorations. The results demonstrate that using enhanced CD90^+^ stem cell populations, cell-based treatments can improve tissue-engineered bone quality in additional craniofacial bone anomalies	Kaigler et al. (2015)
PRP and BMP	PRP and BMP2 on the *in vivo* stimulation of bone formation in an alloplastic substitute were studied. To reconstruct a 10-mm-diameter bone lesion in a rabbit calvarium, biphasic calcium phosphate (BCP) ceramics were combined with PRP, recombinant human BMP2, and their combinations. The bone regeneration of rabbits was studied using radiographic and histomorphometric techniques	Compared to lesions untreated (rhBMP2/BCP without PRP), lesions treated with rhBMP2/BCP combined with PRP had good bone density at 6 and 12 weeks. However, histomorphometrically, the defects filled with rhBMP2/BCP with PRP demonstrated significantly more new bone formation than defects filled with rhBMP2/BCP without PRP, particularly at 6 weeks. The combined effect of PRP and rhBMP2 enhances the osteoinductive potential of alloplastic replacements *in vivo*	Yoshida et al. (2013)
Human iPSCMSCs, human DPSCs	For the first time, researchers have created a novel injectable CPC containing hydrogel fibers encasing stem cells containing hDPSCs, hiPSC-MSCs from bone marrow and foreskin, and hBMSCs for bone engineering	Cell survival was unaffected by injection, whereas CPC injections were 62% porous. = BM-HIPSC-MSCs, BM-hDPSC-MSCs, and hBMSCs show that minerals are produced with time, although not significantly. Cells from the hDPSC, BM-hiPSC-MSC, and hBMSC families are attractive for bone tissue engineering. This new injectable CPC product, which contains cell-encapsulating hydrogel fibers, may help bone regeneration in dental, craniofacial, and orthopedic applications	Wang et al. (2016b)

## 5 Conclusion

The current tissue engineering and RM approaches in face aesthetics surgery and craniofacial restoring and reconstruction offer ways to repair congenital or acquired abnormalities of tissue types, such as nerves, soft tissue, blood vessels, bone, and cartilage. However, significant progress has been achieved, and preclinical and clinical trial results seem promising; discrepancies in some study designs may draw incorrect results, so they cannot be used in clinical experiments. Some research has shown success with TE of cartilage and bone. The early findings suggest that soft tissue can also be successfully regrown. There is a need for some research on scaffold properties, cell types, and delivery techniques in the most efficient way before RM/TE can be used clinically in craniofacial surgery. This will allow more advances and advancements as interdisciplinary cooperation between polymer chemistry, molecular genetics, robotics, molecular biology, mechanical engineering, and materials science gains strength. There are some limitations in damaged tissue or tissue defect healing. The limitation of surgery is that it neither guarantees nor promotes healing. As best as they can, surgeons seek to remove known barriers to healing, including infections, instability, and foreign bodies. However, most studies had enough sample size, and some lacked enough samples to be considered vital. On the other side, however, many studies have been performed on biomaterials and natural materials. All of their effects are not revealed yet, and it is not possible to use them with no caution in preclinical procedures. Cell therapy (especially stem cells) and growth factor usage have been focused on in recent decades, but their full impacts are not discovered either. Therefore, there is a need to conduct more *in vitro* and *in vivo* studies and develop a simulator bioreactor for performing *ex vivo* experiments. More *in vitro* and *in vivo* studies should be performed, and it is one of the most critical limitations regarding RM.

## 6 Future direction

A recent technological advance in craniofacial and dental reconstruction is the 3D printing of biomaterials. Nanotechnology has also been shown to be highly beneficial. TE is potentially replacing more sophisticated template manufacturing procedures with 3D printing, a rapidly evolving technology that uses computer-enabled printers to organize a template into a proper 3D form. In craniofacial restoration, 3D printing may be utilized to fabricate porous materials with more vital interconnections and produce suitable templates for replacing and treating bone defects with precise anatomical forms. A few case reports on humans have shown successful results with few problems. Studies have demonstrated that nanoparticles, nanotubes, and nanofibers can improve mechanical scaffold properties, cellular adhesion, and tissue regeneration. The biomechanical and biochemical capabilities of nanoparticles have been demonstrated in multiple studies, making TE a promising treatment for MF and oral tissues. Nanomaterials may build up in several organs because accurate dose-response and toxicity screening approaches must be developed before regular clinical trials.

## Glossary

3D: Three-dimensional
ADSC: Adipose-derived stem cells
AFD: Acrofacial dysostoses
AFT: Autologous fat transfer
AM: Additive manufacturing
A-PRF: Advanced platelet-rich fibrin
AR: Augmented reality
ARP: Alveolar ridge preservation
AV: Arteriovenous
bFGF: Basic fibroblast growth factor
BGC: Bioactive glass-ceramic
BMMSC: Bone marrow mononuclear cell
BMP: Bone morphogenetic protein
BMSC: Bone marrow stromal cell
CAF: Coronally advanced flap
CaP: Calcium phosphate
CBCT: Cone-beam computed tomography
CFD: Craniofacial defect
CH: Chitosan
CLP: Cleft lip and palate
CMF: Craniomaxillofacial
CMX: Collagen matrix
CPC: Calcium phosphate cement
CSF: Cerebrospinal fluid
CTA: Composite tissue allotransplantation
DM: Diabetes mellitus
DPSC: Dental pulp stem cells
ECM: Extracellular matrix
EGF: Epidermal growth factor
ESC: Embryonic stem cells
FD: Fibrous dysplasia
FFPM: Fractures of the frontal process of the maxilla
FGF: Fibroblast growth factor
FPM: Frontal process of the maxilla
GA: General anesthesia
HA: Hyaluronic acid
HFA: High-frequency acceleration
IGF-I: Insulin-like growth factor-I
IL: Interleukin
iPSC: Induced pluripotent stem cell
MBCaP: Macro biphasic calcium phosphate
MF: Maxillofacial
MMP: Matrix metalloproteinase
MSC: Mesenchymal stem cell
MTA: Mineral trioxide aggregates
NGF: Nerve growth factor
PDLSC: Periodontal ligament stem cell
OPG: Osteoprotegerin
OPMD: Oral potentially malignant disorders
PCL: Poly(ε-caprolactone)
PDMS: Polydimethylsiloxane
PLAGA: Poly(lactic-co-glycolic acid)
POH: Progressive osseus heteroplasia
PPF: Poly(propylene fumarate)
PRF: Platelet-rich fibrin
PRP: Platelet-rich plasma
PTE: Potentially traumatic event
RSV: Resveratrol
SCAP: Stem cells from apical papilla
SHED: Stem cells from human-exfoliated deciduous teeth
TBF: Temporal bone fracture
TCA: Trichloroacetic acid
TE: Trace element
TRC: Tissue repair cell
TSPSC: Tissue-specific progenitor stem cell
VEGF: Vascular endothelial growth factor
VSP: Virtual surgical planning
WHO: World Health Organization.
